# Peptide-based positron emission tomography probes: current strategies for synthesis and radiolabelling

**DOI:** 10.1039/d2md00397j

**Published:** 2023-01-06

**Authors:** Mariacristina Failla, Giuseppe Floresta, Vincenzo Abbate

**Affiliations:** a Department of Drug Science and Technology, University of Turin Via P. Giuria 9 10125 Turin Italy; b King's College London, Institute of Pharmaceutical Science Franklin Wilkins Building London SE1 9NH UK giuseppe.floresta@unict.it vincenzo.abbate@kcl.ac.uk; c Department of Drug and Health Sciences, University of Catania Catania Italy

## Abstract

In medical imaging, techniques such as magnetic resonance imaging, contrast-enhanced computerized tomography, and positron emission tomography (PET) are extensively available and routinely used for disease diagnosis and treatment. Peptide-based targeting PET probes are usually small peptides with high affinity and specificity to specific cellular and tissue targets opportunely engineered for acting as PET probes. For instance, either the radioisotope (*e.g.*, ^18^F, ^11^C) can be covalently linked to the peptide-probe or another ligand that strongly complexes the radioisotope (*e.g.*, ^64^Cu, ^68^Ga) through multiple coordinative bonds can be chemically conjugated to the peptide delivery moiety. The main advantages of these probes are that they are cheaper than classical antibody-based PET tracers and can be efficiently chemically modified to be radiolabelled with virtually any radionuclide making them very attractive for clinical use. The goal of this review is to report and summarize recent technologies in peptide PET-based molecular probes synthesis and radiolabelling with the most used radioisotopes in 2022.

## Abstract 2

In medical imaging, techniques such as magnetic resonance imaging, contrast-enhanced computerized tomography, and positron emission tomography (PET) are extensively available and routinely used for disease diagnosis and treatment.

## Graphical abstract

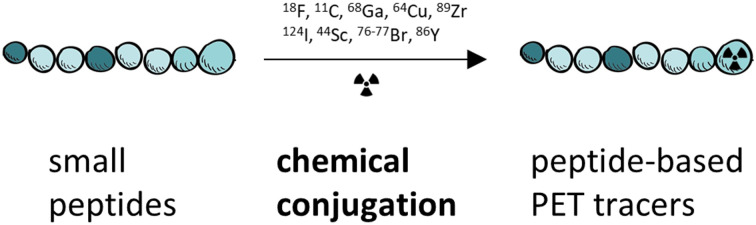

## Introduction

Radionuclides are radioactive isotopes of elements that can be exploited for applications in medical imaging, as well as for cancer therapy.^1^ DOTATOC, a somatostatin receptor-targeted ligand, was firstly approved for diagnostic and therapeutic purposes in patients with somatostatin-expressing neuroendocrine tumours more than two decades ago and it paved the way for other clinical applications of similar analogues with peptides and peptide-like chemical structures.^2^ Since the approval of DOTATOC, impressive improvements have been made in the development of novel radiopharmaceuticals for theranostic applications. A big effort is currently being made in the design of novel radiolabelled peptide-based probes with favourable biodistribution and binding to target structures in living cells and tissues, *e.g.*, enzymes, cellular transporters, or membrane receptors.^3–5^ The application of these probes allows focused diagnostic and targeted therapy in several fields such as cardiology, endocrinology, and neurology, to investigate infection and inflammation and most importantly in oncology. Because of the increasing global incidence of malignant tumours in the population, this area of application results of particular interest. In cancer cells, the interactions of the radiolabelled peptide-based probe are normally mediated through high-affinity binding with specific protein structures overexpressed in these malignant cells.^6,7^ To exploit these peptides as imaging or therapeutic agents, they must be covalently or non-covalently radiolabelled. The radiolabelling of peptides can be performed by direct labelling with the radionuclide (covalent bond), or by using chelators, and the therapeutic or diagnostic capability is driven by the nature of the selected radionuclide for the radiolabelling of the peptide.^8^ Covalent bonds can be used to label peptides without using intermediates and modify the molecular structure of the peptide and the process can also be optimized to a one-step protocol, which is an advantage for kit-based radiopharmaceuticals and short-lived radionuclides. An example of this practice is the one-step direct ^18^F labelling and the most modern radioiodination. On the other hand, radiometal ions such as ^67^Ga^3+^ and ^68^Ga^3+^ must be caged from an aqueous solution using appropriate chelator agents.^1^ The aim of this review is to summarize and report the most significant outputs related to the development of novel synthetic approaches for covalent and non-covalent radiolabelling of radiopharmaceuticals with peptide structure for PET-based medical applications. The discussed radionuclides are summarized in Table 1.

**Table 1:** Radionuclides covered in this review

Isotope	Half-life	Decay mode (% positron emission)
^18^F	109.8 min	β+ (97%)
^11^C	20.4 min	β+ (100%)
^68^Ga	67.7 min	β+ (87%)
^64^Cu	12.7 h	β+ (17%)
		β− (39%)
		EC (44%)
^89^Zr	3.3 d	β+ (23%)
		EC (77%)
^124^I	4.2 d	β+ (23%)
		EC (77%)
^44^Sc	4.04 h	β+ (100%)
^76^Br	16.2 h	β+ (54%)
		EC (46%)
^77^Br	57 h	β+ (100%)
^86^Y	14.7 h	β+ (100%)

## Design and strategies for ^18^F marked peptides

**Fig. 1:**
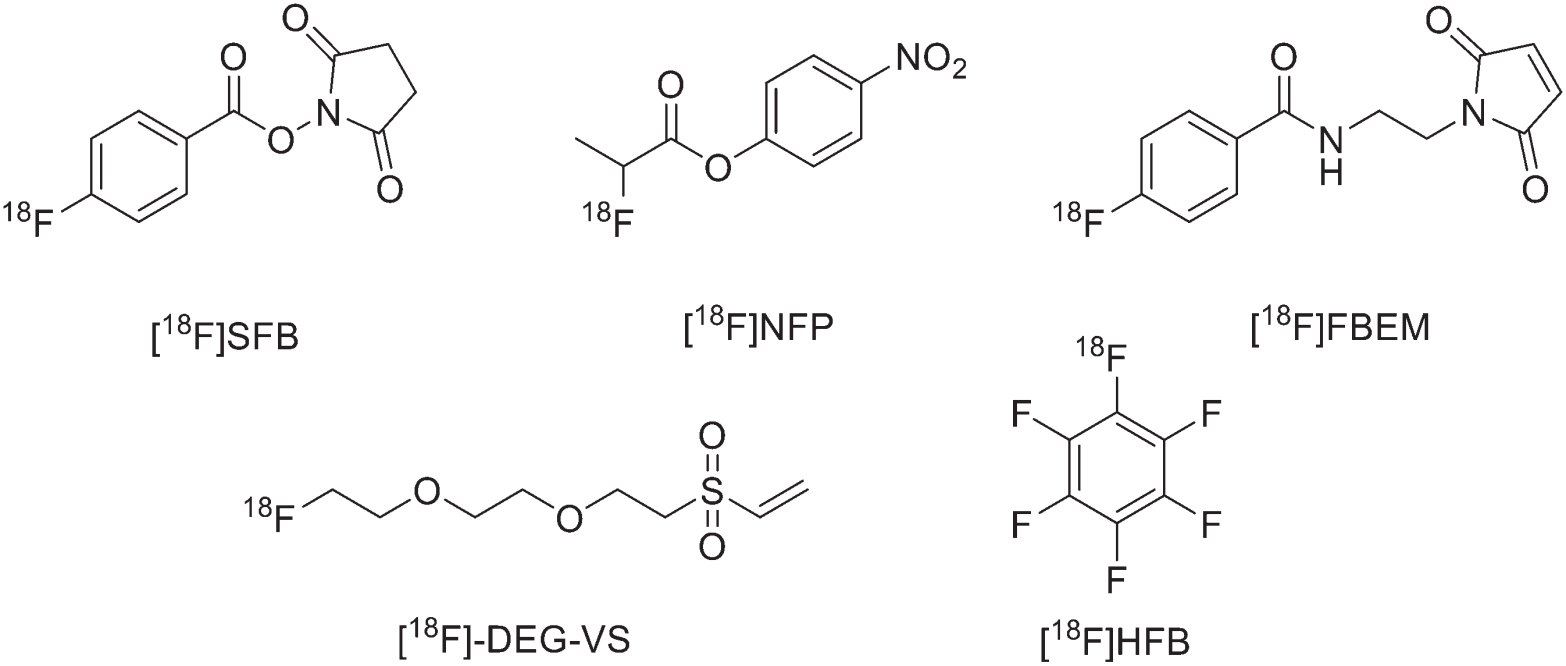
Examples of most recently applied ^18^F-labelled prosthetic groups connected to peptides for PET.

^18^F was the most largely used radioisotope for peptide-based tracers due to its ready availability, half-life of 110 min and high% positron decay. However, direct fluorination of peptides could be hard due to the conditions normally required (not aqueous media). For this reason, indirect labelling is usually employed through conjugation of ^18^F labelled prosthetic groups, on which, at first, ^18^F is inserted and then conjugated to the peptide. The most recently employed prosthetic groups are illustrated in Fig. 1.

The main derivative is *N*-succinimidyl-4-[^18^F]fluorobenzoate ([^18^F]SFB), (Fig. 1). This was described by Vaidyanathan and Zalutsky^9^ in 1994. Later, Wester *et al.* improved the synthesis by using TSTU as activating agent; this method reduced the radiosynthesis time and eliminated the final step of HPLC purification.^10^ More recently, Zhang *et al.* developed two new ^18^F-labelled radiotracers containing Ac-TC14012, a peptide with high affinity for CXCR4, which belongs to the chemokine receptors family and is overexpressed in tumour cells from many types of human tumours.^11^ The peptide was labelled on Lys.^7^ 4-Nitrophenyl 2-[^18^F]-fluoropropionate ([^18^F]NFP) and ([^18^F]SFB) (Fig. 1) were prepared on a GE TRACERLab FXN module and on an Eckert and Ziegler (Eurotope GmbH) module, respectively, as previous reported.^12,13^ Subsequently, for the synthesis of [^18^F]FP-Ac-TC14012, [^18^F]NFP and Ac-TC14012 in DMSO were reacted in presence of DIPEA at 80 °C for 10 min. When the reaction was complete, the mixture was cooled and diluted with water containing 0.1% TFA and purified by semi-preparative HPLC. For the synthesis of [^18^F]FB-Ac-TC14012, the peptide and [^18^F]SFB were reacted in DMSO and 100 mM Na_2_HPO_4_ buffer, pH 8.5, was added to the tube and reacted at RT for 20 min. At the end of the reaction, the final compound was obtained as described above. The radiochemical yield (RCY) for coupling of [^18^F]NFP with Ac-TC14012 was 38.1 ± 10.5%, while the yield for coupling of [^18^F]SFB with Ac-TC14012 was 31.6 ± 7.0%. The radiochemical purities (RCPs) were ≥95% for both compounds. The specific activity ranged from 13.6 to 17.6 GBq μmol^−1^ (at the time of measurement) for [^18^F]NFP-labelled peptide and from 18.7 to 31.6 GBq μmol^−1^ for [^18^F]SFB derivative. The [^18^F]FP-labelled peptide displayed higher tumour uptake, lower non-specific binding, and better tumour-to-background contrast with respect to the [^18^F]FB analogue.

The use of maleimide derivatives as prosthetic groups exploits the chemoselectivity of thiol groups of amino acids like Cys. For example, Kiesewetter *et al.* synthesised ^18^F radioligands for GLP-1R by the reaction of the maleimide prosthetic group [^18^F]FBEM (N-[2-(4-[^18^F]fluorobenzamido)ethyl]maleimide, Fig. 1), with [Cys0] and [Cys40] analogues of exendin-4.^14^ Exendin-4, which does not have any cysteine residues in the native structure, was modified with either a C-terminal or N-terminal cysteine to allow site-specific labelling with [^18^F]FBEM. For the synthesis of [^18^F]FBEM, 4-[^18^F]fluorobenzoic acid was, at first, obtained by nucleophilic substitution of a trimethylammonium moiety on a pentamethylbenzyl benzoate ester with [^18^F]fluoride. Subsequently, the ester was hydrolysed under acidic conditions. Finally, 4-[^18^F]fluorobenzoic acid was coupled to *N*-(2-aminoethyl)maleimide using diethylcyanophosphate and DIPEA. After HPLC purification, [^18^F]FBEM was obtained as a solution in DCM in 18.8 ± 3.1% radiochemical yield, an improvement with respect to work by Cai *et al.*^15^ in terms of radiochemical yield and number of synthetic steps. After that, the right amount of [^18^F]FBEM solution (range 388.5–984.2 MBq) was evaporated to near dryness and the tube was vortexed after the addition of EtOH. [Cysx]-Exendin-4 in 0.1% sodium ascorbate in PBS was added, and the solution was vortexed followed by incubation for 30 min. Aqueous 0.1% TFA was added, and the solution was injected into a semipreparative HPLC which allowed the separation between the desired compounds from the radioactive oxidation product, the unreacted [Cys0]- or [Cys40]-exendin-4 and the disulfide dimer of [Cys0]- or [Cys40]-exendin-4. Radiochemical purity was >96%. The radiochemical yields for [^18^F]FBEM-[Cys0]-exendin-4 and [^18^F]FBEM-[Cys40]-exendin-4 were 34.3 ± 3.4% without difference between the two isomers. The average specific activity of all compounds was 45.51 ± 16.28 GBq μmol^−1^ (1.23 ± 0.44 Ci μmol^−1^; *n* = 7) at the time of measurement at the end of the radiochemical synthesis. [^18^F]FBEM-[Cys40]-exendin-4 displayed higher tumour uptake than the [Cys0] isomer, representing a potential good candidate as a GLP-1R-imaging agent. The employment of labelled maleimide derivatives for reactions with peptides presents the challenge of their high synthetic complexity and results in species that exist as two stereoisomers. These conjugates are easily hydrolysed at neutral pH, and, in addition, a multi-step synthesis is often required, with consequent low yields. To overcome this issue, Wu *et al.* synthesised a fluorinated vinyl sulfone (VS) through a one-step ^18^F-fluorination and conjugated it to free thiol groups in thiolated neurotensin (NT) peptide.^16^

**Scheme 1:**
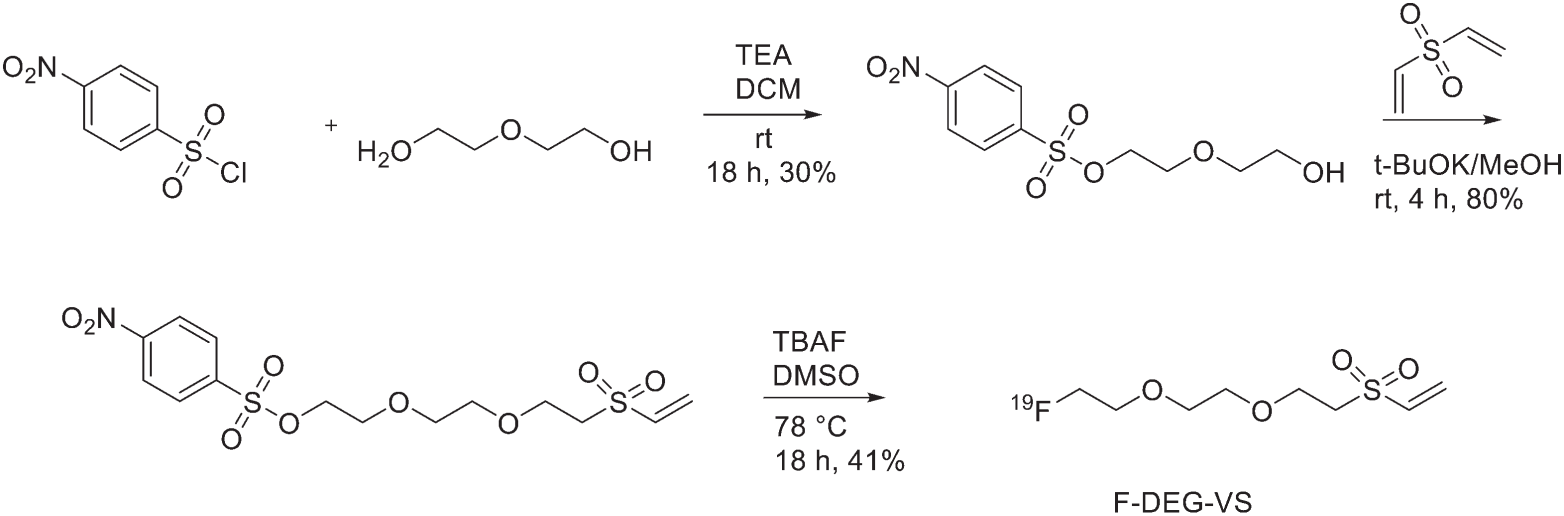
Synthetic scheme of F-DEG-VS.

As reported in Scheme 1, [2-(2-(2-(vinylsulfonyl)ethoxy)ethoxy)ethyl]4-nitrobenzenesulfonate was synthesised in 20% yield after two steps starting from 4-nitrobenzene sulfonyl chloride and diethylene glycol and then divinylsulfone. It was ^19^F-fluorinated by reacting with TBAF at 78 °C overnight. The reaction mixture was purified by HPLC to give the desired compound as a yellowish liquid (yield 41.3%). For the synthesis of ^18^F-(2-(2-(2-fluoroethoxy)ethoxy)ethylsulfonyl)ethane (^18^F-DEG-VS), the ^18^F–fluoride solution was eluted from the QMA cartridge and dried with consecutive MeCN evaporations at 90 °C. A solution of F-DEG-VS in DMSO was added to the reactor and heated at 80 °C for 10–15 min. Acetic acid was added to quench the reaction. Then, ^18^F-DEG-VS and borate buffer (pH 8.5) were added into neurotensin or (Ac)-NT and the reaction mixture was incubated at RT for 5–10 min. The reaction was quenched by acetic acid (5%) and the product was purified by radio HPLC (radiochemical purity >98%, specific activity 19.2 ± 4.3 TBq mmol^−1^ and 95% labelling yield). The selectivity of ^18^F-DEG-VS for thiols in the presence of a free amino group was also demonstrated by reacting ^18^F-DEG-VS and c(RGDyC) (with a free thiol group) and c(RGDyK) (with a free amino group) peptides (RGD: Arg–Gly–Asp): only the product from c(RGDyC) and ^18^F-DEG-VS was obtained and even at pH 8.5, ^18^F-DEG-VS selectively labelled the SH in the presence of an NH_2_, both in RGDs peptides and in the NT analogue. The obtained neurotensin receptor-targeted PET agent demonstrated high tumour-to-background contrast and compared with ^18^F-FBEM-NT, it was not only synthesised with few steps but also demonstrated higher *in vivo* imaging quality. Another thiol-reactive prosthetic group is [^18^F] hexafluorobenzene ([^18^F]HFB, Fig. 1). HFB reacts with free SH to give a perfluoroaromatic connection between two sulfurs. Jacobson *et al.* prepared ^18^F-HFB by a fluorine exchange reaction using K^18^F/K_2.2.2_ at RT. ^18^F-HFB was collected by distillation and then reacted with thiolated c(RGDfk) peptide in a basic and reducing environment. The product was isolated in 40 ± 2% radiochemical yield (*n* = 4) with a specific activity of 15 mCi μmol^−1^. The ^18^F-RGD-TFB-RGD exhibited integrin receptor specific binding, good cellular uptake, and *in vivo* tumour accumulation.^17^

### Click chemistry

**Scheme 2:**
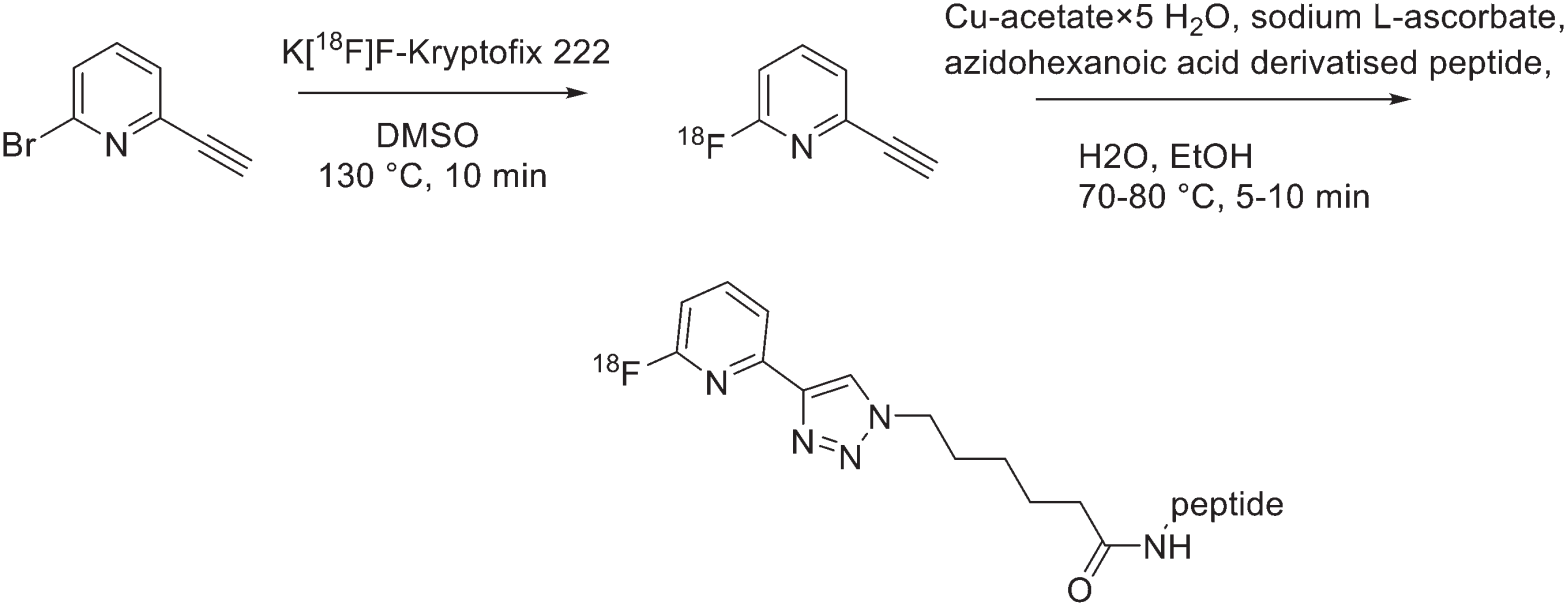
Synthetic scheme for radiolabelled products.

Recently, click chemistry, the 1,3-dipolar cycloaddition of an azide and an alkyne at RT, was also introduced for ^18^F-labelling. Both ^18^F-labelled alkynes and azides have been employed as prosthetic groups for the labelling of modified peptides. For example, Daumar *et al.* developed the 6-[^18^F]fluoro-2-ethynylpyridine prosthetic group to be linked *via* click chemistry to azidohexanoic acid derivatised pH low insertion peptide (pHLIP). It is a 37-residue pH insertion peptide able to insert into cell membranes at a low extracellular pH.^18^ This prosthetic group was synthesised *via* nucleophilic displacement of 2-bromo-6-ethynylpyridine with radiochemical purity >98%, radiochemical yields 27.5 ± 6.6% (*n* = 11) and conversion rates between 70% and 90% (Scheme 2). The subsequent copper(i)-catalysed azide–alkyne cycloaddition (CuAAC) with azido-functionalised pHLIP peptides was completed within 5 min at 70 °C (quantitative yields).

**Scheme 3:**
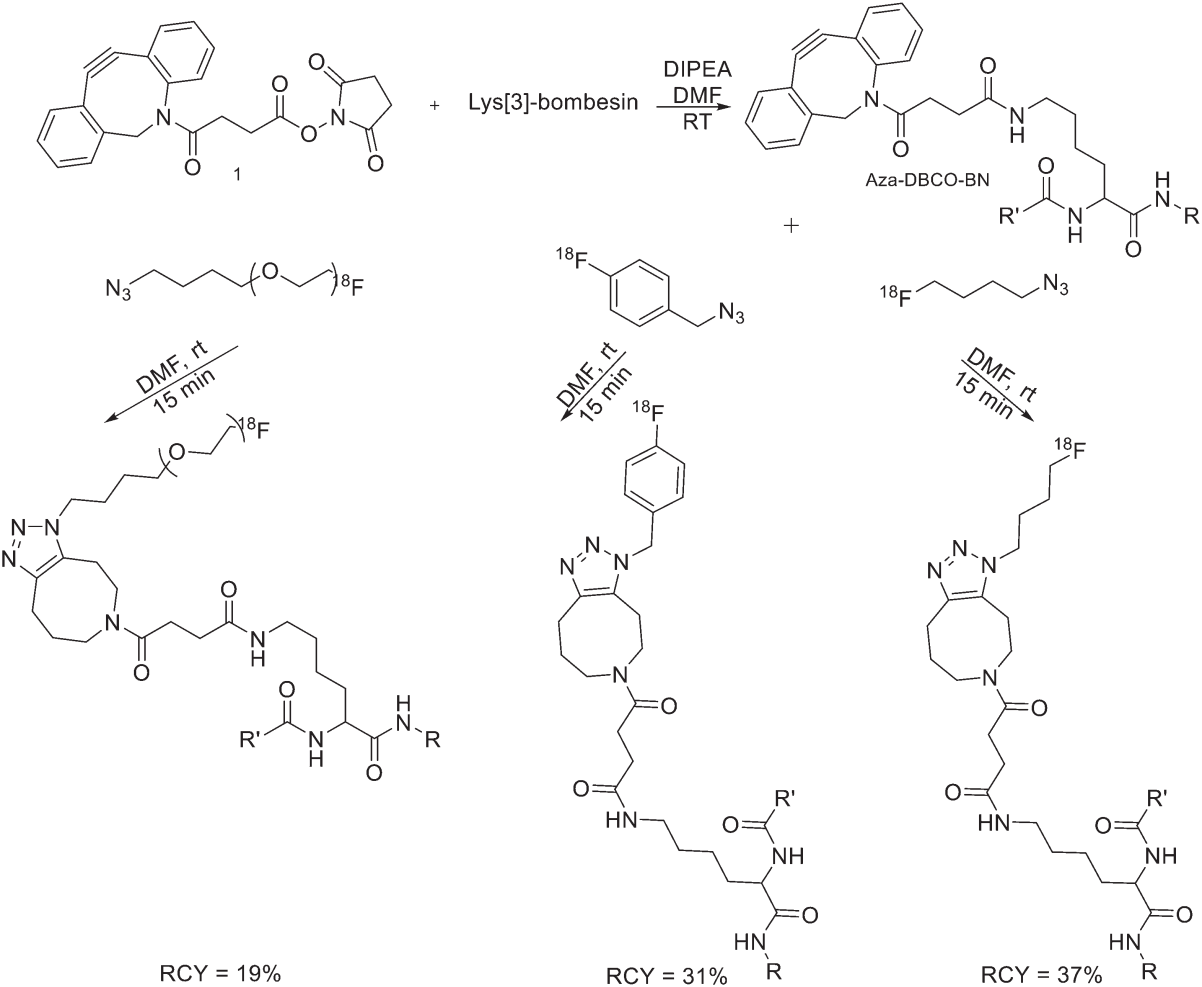
Synthetic scheme for the preparation of the three different ^18^F-bombesin analogue peptides. R = Pyr–Gln; R′ = Leu–Gly–Asn–Gln–Trp–Ala–Val–Gly–His–Leu–Met–NH_2_, Pyr = pyroglutamic acid.

Since it was not possible to separate the radiolabelled peptides from the starting azido-derivatised materials, the apparent specific activities were low (0.1–1 GBq μmol^−1^). This should not represent a problem for the use of [^18^F]-pHLIPs as efficient PET tracers. In fact, given the pHLIP transmembrane insertion mechanism, when the microenvironment is acidic, the insertion sites are theoretically infinite. One of the two labelled peptides showed good *in vitro* stability, and only moderate *in vivo* defluorination was observed in both cases. Campbell-Verduyn *et al.* synthesised a Lys3-BBN modified with a strained alkyne to allow fast labelling with ^18^F without the presence of copper.^19^ After the synthesis of *N*-hydroxysuccinimide ester 1 (Scheme 3) starting from commercially available dibenzosuberenone, it was conjugated (5 equiv.) to Lys[3]-bombesin in presence of DIPEA (10 equiv.) in DMF at RT. Conversion to the final Aza-DBCO-BN was completed after 24 h and after that, it was purified by RP-HPLC. Then, it was tested the efficiency of the [3 + 2] cycloaddition in absence of copper with various ^18^F-containing azides. Reaction between the radionuclide-containing azide with the modified bombesin analogue for 10–15 min at RT gave the target peptides in modest to good yields. The specific activities were 62 GBq μmol^−1^, 57 GBq μmol^−1^, and 60 GBq μmol^−1^ respectively. Despite the modification, all the PET tracers maintained a high affinity for the GRPRs.

### Aluminium-fluoride radiofluorination

**Scheme 4:**
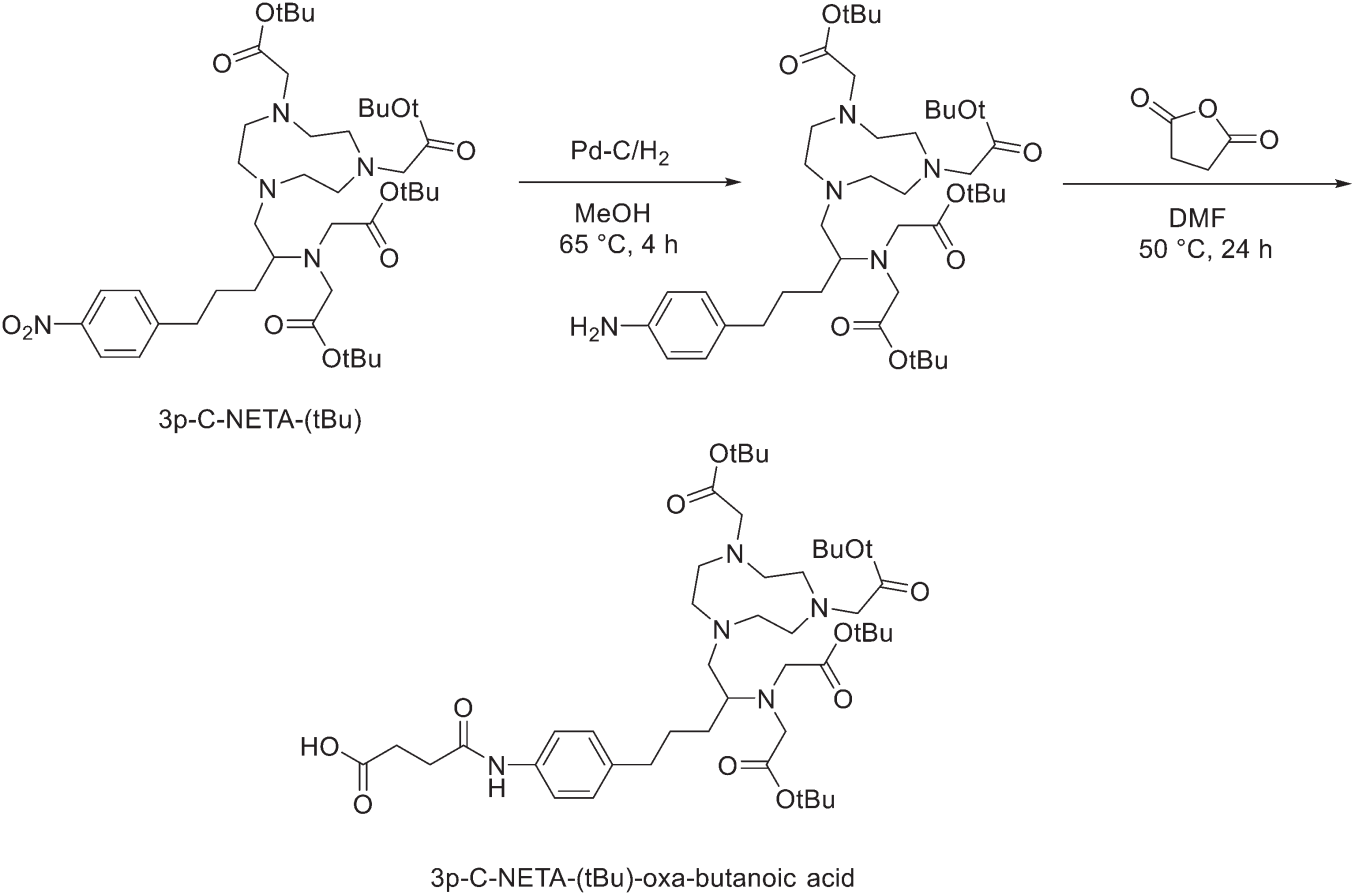
Synthetic route for 3p-*C*-NETA-(*t*Bu)-oxa-butanoic acid.

Another strategy for the direct ^18^F labelling of peptides consists in the introduction of aluminium fluoride (AlF) species. It is well known that fluorine can strongly interact with different metals, *e.g.*, aluminium, and this strong interaction gave birth to a new class of ^18^F-labelled peptides. Al^3+^ can form an octahedral complex that is perfectly suited to pentadentate chelation systems such as diethylenetriamine pentaacetic acid (DTPA) or 1,4,7-triazacyclononane-1,4,7-triacetic acid (NOTA). AlF^2+^ chelation to NOTA is achieved in good RCY in approximately 15 min at 100 °C.^20^ With the Al^18^F-technique, imaging and logistical advantages of ^18^F are combined with the benefits of a chelator-based radiolabelling method. [^18^F]AlF-NOTA-octreotide and [^18^F]AlF-FAPI-74 are some excellent examples of this promising development.^21–23^ Recently, Ahenkorah *et al.*^24^ found an alternative to NOTA as Al^18^F-chelator that can also be used in combination with therapeutic radionuclides (^177^Lu, ^161^Tb, ^213^Bi, ^225^Ac and ^67^Cu), and has superior labelling kinetics over DOTA. 3p-*C*-NETA ({4-[2-(bis-carboxy-methylamino)-5-(4-nitrophenyl)-entyl]-7-carboxymethyl-[1,4,7]tri-azonan-1-yl}acetic acid), a ligand bearing both a parent macrocyclic NODA (1,4,7-triazacyclononane-*N*,*N*′-diacetic acid) backbone and a flexible acyclic tridentate pendant arm, has been reported by Chong *et al.* to be a promising chelator in terms of kinetics and stability for β^−^emitters, such as ^90^Y and ^177^Lu.^25–27^ As 3p-*C*-NETA also contains a NODA backbone, Ahenkorah and co-workers^24^ assumed that it might be a suitable chelator for Al^18^F-chelation and thus could be a good theranostic chelator for ^18^F and therapeutic radionuclides. They synthesised the 3p-*C*-NETA, based on a published method developed by Kang *et al.*,^28^ and its new bifunctional derivative 3p-*C*-NETA-(*t*Bu)-oxa-butanoic acid. Then, they evaluated the radiolabelling properties of 3p-*C*-NETA with a range of therapeutic (^213^Bi, ^161^Tb, ^177^Lu, ^67^Cu, and ^225^Ac) and diagnostic radionuclides including Al^18^F. To enable conjugation of 3p-*C*-NETA to a vector molecule, 3p-*C*-NETA was functionalised initially with isothiocyanate as reported by Kang *et al.*^28^ However, conjugation to the (Tyr^3^)-octreotate moiety by forming a thiourea bond resulted in many undesired side products. Therefore, they changed the functionalisation strategy by introducing succinic acid to form 3p-*C*-NETA-(*t*Bu)-oxa-butanoic acid to enable efficient conjugation *via* amide bond formation (Scheme 4). For the conjugation, 2 equiv. of peptide were dissolved in 500 μL of DMF and 10 μmol of 3p-*C*-NETA-(*t*Bu)-oxobutanoic acid, 2 equiv. of HATU and DIPEA were added to the solution. The reaction was monitored by LC–MS. Upon completion, 15 μL of hydrazine monohydrate were added to the solution (for a final concentration of 2% hydrazine in DMF). IvDde deprotection was monitored by LC–MS and quantitative deprotection was obtained in 15 min. The reaction mixture was concentrated *in vacuo* and then purified by preparative HPLC to yield the *tert*-butyl protected compound (>95% purity) as a white solid in 37% yield. *Tert*-butyl deprotection was performed by treatment of the compound with 1 mL of a solution of TFA/DCM/TES (v/v/v = 44 : 44 : 2) for 5 h. The mixture was concentrated under nitrogen flow and the product was purified by preparative HPLC to yield the desired compound 3p-*C*-NETA-TATE with purity >95% and a yield of 56%. The macrocyclic hexadentate ligand NOTA and especially the pentadentate derivative NODA remains the gold standard for Al^18^F-labeling of heat-stable molecules, but high temperatures (95 °C) are needed to obtain good radiochemical conversions (RCCs). 3p-*C*-NETA has the same macrocyclic core (triazacyclononane backbone) as NODA, but has in addition a flexible acyclic tridentate pendant arm (iminodiacetic acid) which was expected to improve the reaction kinetics to coordinate [^18^F]AlF compared to NODA. However, only at 95 °C, a good RCC of 62.3 ± 1.5% was obtained. It has been reported before, that performing the Al^18^F-reaction at lower ionic strength by addition of co-solvents (*i.e.* ethanol, acetonitrile), increased RCCs considerably.^29^ Accordingly, a reaction content of 50% absolute ethanol increased the RCC at 95 °C to 80.3 ± 0.6%, and this is comparable to the [^18^F]AlF-labeling of NODA-benzyl.^30^ 3p-*C*-NETA-TATE was radiolabelled with [^18^F]AlF in an automated AllinOne® synthesis module ([^18^F]AlF-NOTA-octreotide method)^31^ and the corresponding [^18^F]AlF-3p-*C*-NETA-TATE was obtained in good RCY [41.4 ± 8%, (decay-corrected, activity final batch of purified product/activity in reactor, *n* = 8, apparent molar activity 22 ± 8 GBq μmol^−1^)] and an excellent radiochemical purity (>97%). Using identical radiolabelling conditions, the RYC was even better than for [^18^F]AlF-NOTA-octreotide (RCY of 26.3 ± 3.6%),^32^ illustrating the robustness of the developed automated Al^18^F-radiolabelling protocol and indicating that 3p-*C*-NETA was indeed a promising chelator for the Al^18^F-method. As a note, the starting activity of [^18^F]F^−^ was relatively low for the synthesis of [^18^F]AlF-3p-*C*-NETA-TATE used for these preclinical studies, resulting in a moderate apparent molar activity of 22 GBq μmol^−1^, which might have an effect on biodistribution and uptake in SSTR2-expressing organs. The same was observed for [^18^F]AlF-NOTA-octreotide (25 ± 7 GBq μmol^−1^), but for clinical productions the apparent molar activity of [^18^F]AlF-NOTA-octreotide was 160 GBq μmol^−1^ by increasing the starting activity of [^18^F]F^−^. Therefore, similar increase of apparent molar activity for [^18^F]AlF-3p-*C*-NETA-TATE was also expected for future clinical productions.

### Direct labelling

**Scheme 5:**
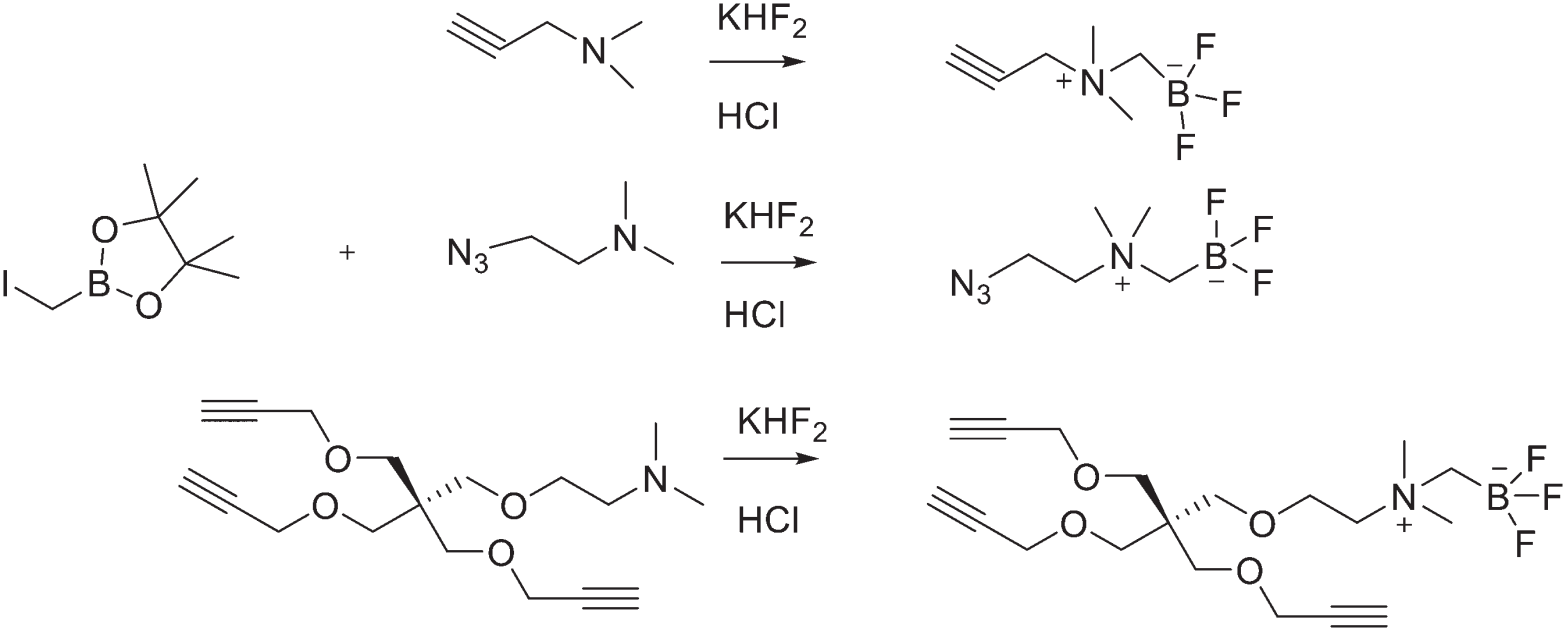
Synthesis of radiosynthons *via* amine alkylation with iodomethylboronylpinacolate followed by conversion to the zwitterionic AMBF_3_.

**Scheme 6:**
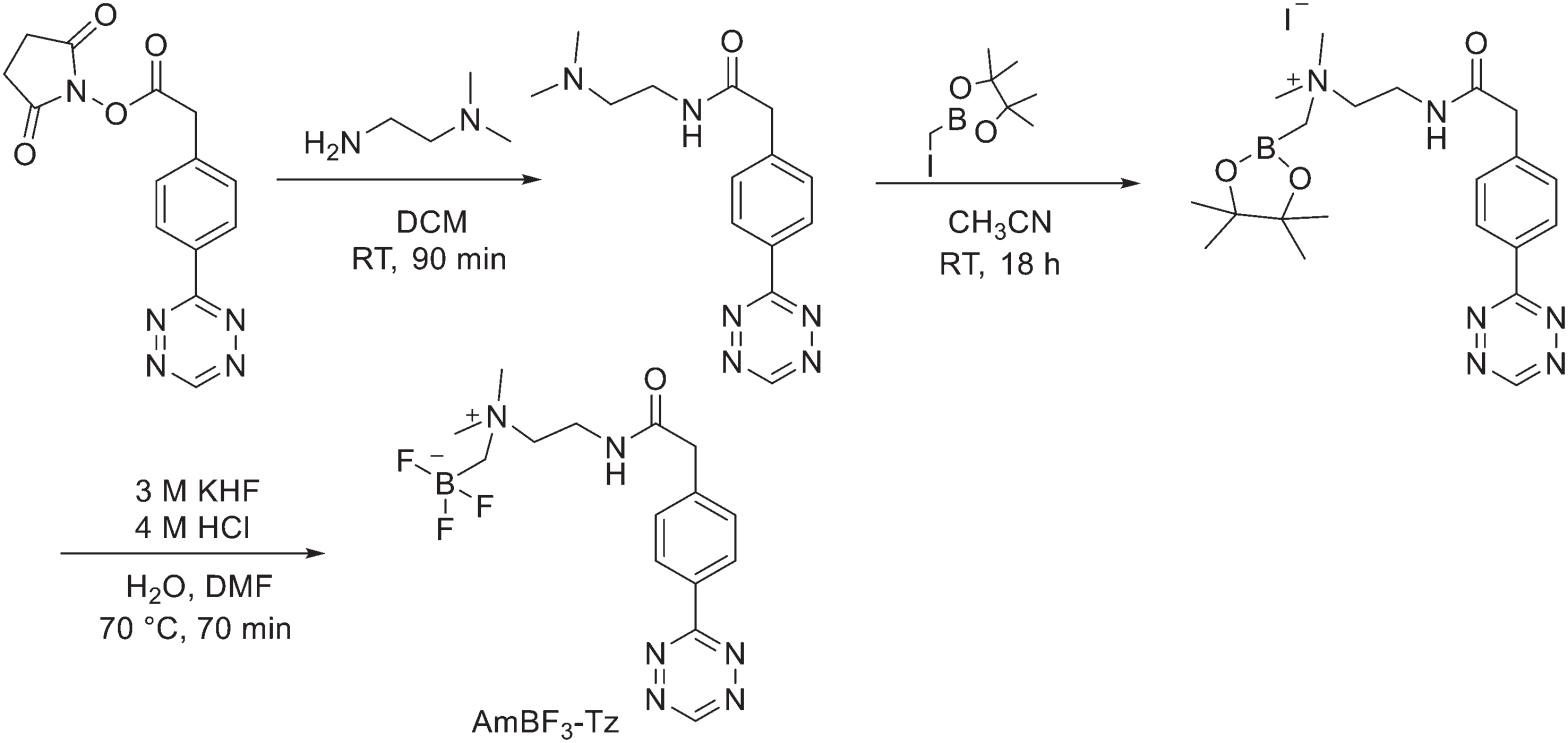
Synthesis of AmBF_3_-Tz.

**Fig. 2:**
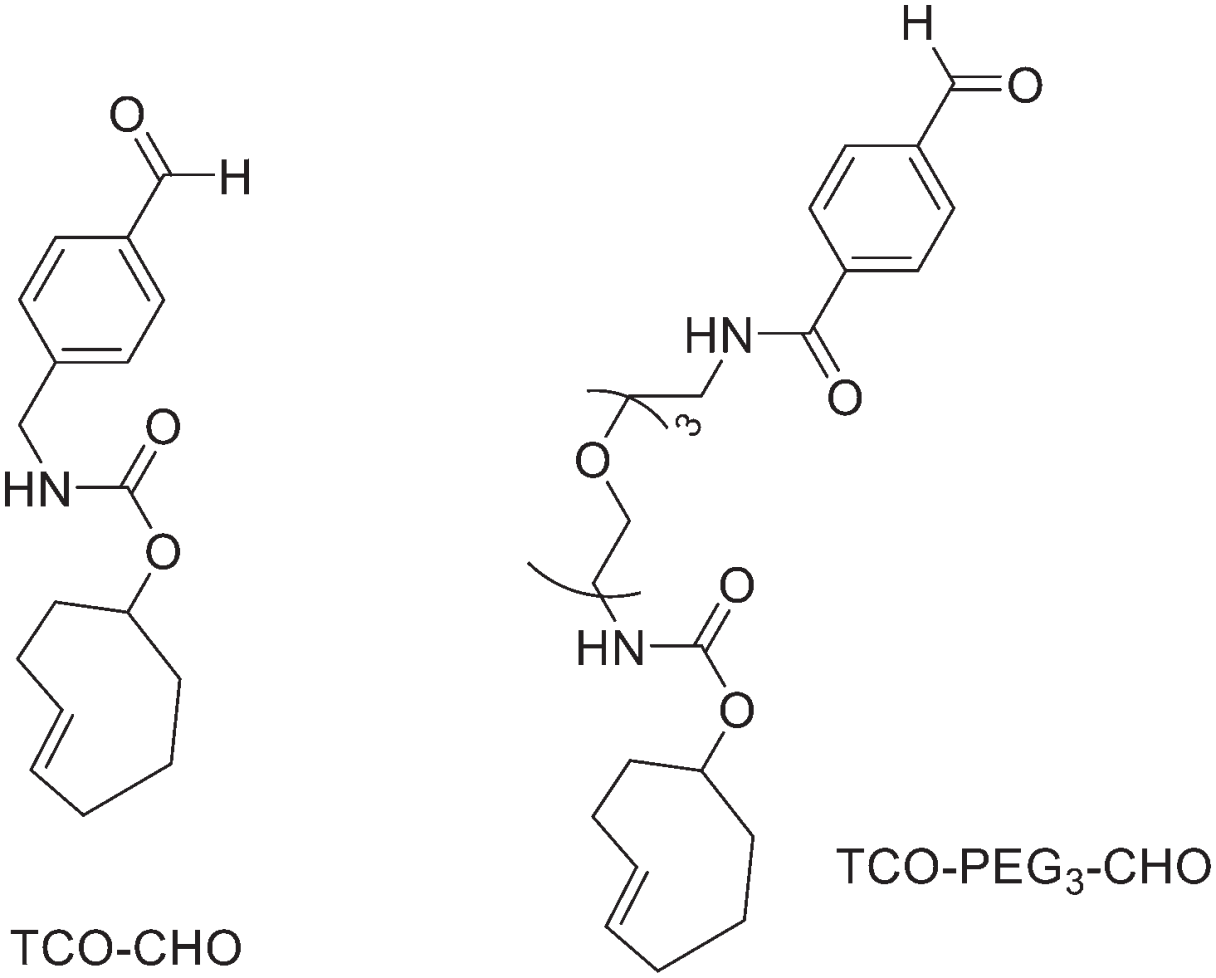
Chemical structures of TCO compounds: TCO–CHO, TCO–PEG_3_–CHO.

Direct labelling requires the direct incorporation of ^18^F on the target peptide in one quick step. The well-known fluorobenzoic acid (FBA) prosthetic group chemistry allowed the development of the first class of direct-labelling groups. FBA could be linked to a free amino group, with a specific leaving group in the *para*-position and an electron-withdrawing group in the *meta*-position. For example, Becaud *et al.*^33^ described the direct labelling of peptides through ^18^F–fluoride nucleophilic aromatic substitution employing trimethylammonium as a leaving group. More recently, Jacobson *et al.* reported a new single-step labelling method with ^18^F–fluoride by replacing a nitro group in an arene with an *ortho* trifluoromethyl group, thus activated toward nucleophilic aromatic substitution. They applied this labelling method to cyclic RGD peptide derivatives containing 4-nitro-3-trifluoromethyl arene as precursors for direct ^18^F labelling.^34^ The conjugation between the 4-nitro-3-trifluoromethylbenzoyl chloride with monomeric RGD peptide c(RGDfK) and dimeric RGD peptide E[c(RGDfK)]_2_ took place by dissolving the peptide (8–10 mg) in DMF. Then, 1.2 equiv. of 4-nitro-3-trifluoromethylbenzoyl chloride was added, followed by the addition of 10 equiv. of triethylamine. The reaction mixture was stirred at RT for several hours. Purification of the two compounds was conducted on an RP-HPLC. ^18^F–Fluoride displacement of the NO_2_ was done rapidly by using a microwave at 130 °C and a low amount of peptide precursors. Both ^18^F-labelled peptides showed radiochemical purity >99%, with high specific activity of 79 ± 13 GBq μmol^−1^. The total radio-synthesis time was approximately 40 min. However, direct labelling *via* nucleophilic substitution was not a method suitable for more complex and temperature-sensitive peptides. Moreover, all the methods described above required one or more purification steps. The most important direct labelling progress has been made thanks to the isotopic exchange (IEX) of ^19^F–^18^F. IEX procedures have been re-evaluated due to the growth of boron–[^18^F]fluorine and silicon–[^18^F]fluorine methodologies. The exploitation of the –BF_3_ functional group for [^19^F–^18^F]-IEX has led to high radiochemical yields one-step radiosynthesis protocols in aqueous conditions. In 2013, Liu *et al.* labelled [^18^F]-aryltrifluoroborate bioconjugates at very high specific activity and good radiochemical yields in 15 min.^35^ However, some complications have arisen, such as the need for HPLC purification and the not optimal *in vivo* stability. To solve these problems, they recently reported a new zwitterionic alkylammoniomethyltrifluoroborate (AMBF_3_), which offers better features for radiotracers' development.^36^ For the synthesis of propargyl-AMBF_3_, azidoethyl-AMBF_3_ and tris-propargylether-AMBF_3_, as shown in Scheme 5, iodomethylboronyl pinacolate (0.9 equiv.) was treated with three different functionalised *N*,*N*-dimethylamines (1 equiv.) in dry diethyl ether to afford the quaternary ammoniomethylboronyl pinacolates in high yields. Trifluoroborates were obtained after reaction in acidic KHF_2_ (1.4 equiv.). For labelling, the precursors were resuspended in aqueous pyridazine/HCl buffer (pH 2.5). No carrier added (NCA) [^18^F]fluoride ion (0.8–1 Ci), obtained by the bombardment of H_2_^18^O with 18 MeV protons, followed by anion-exchange resin trapping (9 mg, QMA, chloride ion form, washed with 1 mL distilled water), was eluted with PBS (*ca.* 60 μL) into the tube containing precursor. They used rhodamine, octreotate, a somatostatin receptor antagonist, a trimeric carbonic anhydrase inhibitor, a trimeric RGD and dual-mode fluorescent-dimeric RGD as precursors. The mixture was heated at 80 °C for 12 min and then it was quenched by NH_4_OH (5% in H_2_O, 2 mL). The reaction mixture was loaded onto a C18 light cartridge. Radiochemically pure radiolabelled product was then released into a glass vial by elution with 1 : 1 EtOH/saline (0.5 mL) to provide ∼200 mCi radiotracer. Depending on the precursor, the radiochemical yields were between 22 ± 9% and 36 ± 5%. All the compounds were obtained with high radiochemical purity and with specific activity between 2.1–4.1 and 3.0–4.0 Ci μmol^−1^, end of synthesis (EOS). AMBF_3_ showed high stability, in fact, no boronic acid was detected, in contrast to previously reported [^18^F]ArBF_3_ compounds where about 50% of the ArBF_3_ was hydrolysed to the ArB(OH)_2_ during the IEX reaction.^18^F-AMBF_3_ was used by Pourghiasian *et al.* to obtain a radiofluorinated derivative of bombesin ^18^F-AMBF_3_-MJ9, a GRPR-targeting peptide for PET imaging of prostate cancer.^37^AMBF_3_-MJ9 was obtained from an AMBF_3_ conjugated alkyne and azidoacetyl-MJ9 *via* a copper-catalysed click reaction (45% yield). The radiochemical purity and specific activity of ^18^F-AmBF_3_-MJ9 were >99% and 100 ± 32 GBq μmol^−1^, respectively. It showed a good binding affinity for GRPR, high plasma stability, good pharmacokinetics, and excellent tumour-to-background contrasts. Since then, the AmBF_3_-chemistry has been utilised for direct IEX radiolabelling of various other peptides.^38,39^ The last example regards the work of Otaru *et al.*^40^ who developed a novel prosthetic group [^18^F]AmBF_3_ tetrazine ([^18^F]-AmBF_3_-Tz) suitable for the chemoselective radiolabelling of *trans*-cyclooctene (TCO)-modified biomolecules. As a model system, they radiolabelled two Tyr3-octreotides (TOCs), analogues of somatostatin,^41^ in a proof-of-concept study evaluating the influence of the novel prosthetic group on the pharmacokinetics of the well-known peptide analogues *in vivo*. The synthesis of the AmBF_3_ tetrazine was designed in a stepwise manner to incorporate the boronic acid pinacol ester selectively into the tertiary amine, followed by acid-catalysed fluorination of the pinacol ester to afford the trifluoroborate (Scheme 6). AmBF_3_-Tz was synthesised with an overall yield of ∼36%. TCO aldehydes TCO–CHO and the PEG analogue TCO–PEG_3_–CHO (Fig. 2) were conjugated to TOC to obtain the TCO-modified TOCs with a purity ≥99% after HPLC purification. After purification, AmBF_3_-Tz was incubated with TCO-modified TOCs in an aqueous solution for conjugating the TCO peptides with AmBF_3_-Tz by IEDDA cycloaddition: the peptide (1 equiv.) in 600 μL of 0.3 M anilinium acetate buffer (pH 4.6) was mixed with TCO–PEG_3_–CHO (1.5 equiv.) or TCO–CHO (1.5 equiv.) in ∼140 μL of chloroform and added dropwise. After 10 min, the peptide was purified with HPLC, MeCN was evaporated, and the residual water-containing fraction was frozen in a freezer (−80 °C) or with a liquid nitrogen bath. Prosthetic group AmBF_3_-Tz was radiolabelled with a protocol partly based on a methodology developed by Liu *et al.*^28^ [^18^F]AmBF_3_ was obtained with molar activity of 6–39.8 GBq μmol^−1^ from the concentrated [^18^F]fluoride in 15 min at 85 °C. The radiochemical yield (RCY) and radiochemical purity (RCP) 20.8 ± 10.3% (*n* = 7, DCY) and ≥98%, respectively. The crude radiolabelled mixture of [^18^F]AmBF_3_-Tz was used for the radiolabelling of TCO-octreotides without cartridge purification. *Trans*-cyclooctene functionalised peptides (25–50 nmol, 500 μL of water) were added into the radiolabelling reaction mixture (∼10–20 μL) of [^18^F]AmBF_3_-Tz (100–200 nmol) and heated at 60 °C (95 : 5 H_2_O : MeCN). After 20 min, the reaction mixture was diluted with water and purified with two SPE C18 cartridges. The total synthesis time was in an average of 85–102 min. The radiochemical yields for both tracers starting from the prosthetic group [^18^F]AmBF_3_-Tz ranged from 8 to 34%. The decay-corrected RCYs of the radiolabelled TOCs, comprising the production of [^18^F]AmBF_3_-Tz and of the subsequent IEDDA reaction (two steps), starting from [^18^F]fluoride, ranged between approximately 2 and 8%, with the radioactivity obtained at 53–267 MBq, with RCPs of ≥99%, and molar activity range of 1.0–9.4 GBq μmol^−1^. The two novel tracers were evaluated by cell uptake studies and *ex vivo* biodistribution in subcutaneous AR42J rat pancreatic carcinoma tumour-bearing nude mice. The tracer demonstrating superior behaviour *ex vivo*, the [^18^F]AmBF_3_-PEG_7_-TOC, was further evaluated with PET/CT, where the tracer provided clear tumour visualization at 25 min post-injection. Thus, the novel AmBF_3_-Tz demonstrated its potential as a prosthetic group for rapid radiolabelling of biomolecules in mild conditions using bioorthogonal chemistry.

**Fig. 3:**
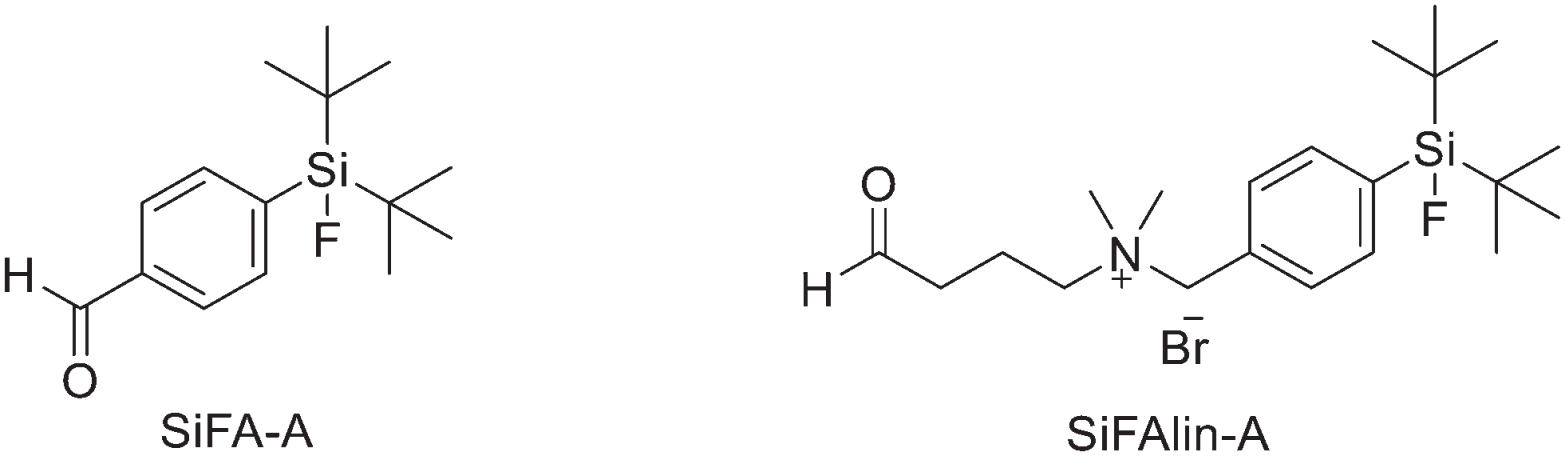
Structures of SiFA and SiFAlin aldehydes.

Another approach for the radiolabelling of peptides with ^18^F is the silicon–fluorine-acceptor (SiFA) methodology. N-Terminally SiFA-modified peptides can be labelled with ^18^F^−^ in a single step at RT without the formation of side products and giving radiochemical yields of 46 ± 1.5%. The discovery that the hydrolysis of the silicon–fluorine (Si–F) bond is minimized when it is surrounded by bulky substituents, led to the first generation of silicon–fluorine acceptors, known as SiFAs. However, the presence of bulky substituents resulted in lipophilic radiopeptides with not optimal *in vivo* characteristics such as mainly hepatobiliary clearance (as demonstrated by the high accumulation of the radiotracers in liver, gallbladder and intestine) and low and unspecific tumour uptake.^42^ To improve the hydrophilicity of these modified peptides, hydrophilic auxiliaries on the SiFA and SiFAlin scaffolds have been introduced (Fig. 3).

For example, Wängler *et al.* introduced hydrophilic moieties such as carbohydrates and polyethylene glycols (PEGs) into the peptide sequence in order to obtain Tyr^3^-octreotate SiFA derivatives with lower lipophilicity.^43^ They synthesised new SiFA-octreotate analogues derivatised with Fmoc–NH–PEG–COOH, Fmoc–Asn(Ac_3_AcNH-β-Glc)–OH, and SiFA-aldehyde (SIFA-A). The SiFA moiety has been introduced through a chemoselective reaction between the aminooxy-derivatised octreotate derivatives and the aldehyde group of SiFA-A to give an oxime, in a mixture of phosphate buffer and MeCN. The coupling reaction took place at pH 4.0 giving the final compounds after only 5 min at RT. In particular, the overall yields were 13.3% and 15.2% after final HPLC purification, respectively. No radioactive side products were observed, apart from unreacted ^18^F, thanks to the mild conditions employed for the SiFA-isotopic exchange labelling. When starting from stock solutions of dried [^18^F]F-/Kryptofix 2.2.2/K^+^ complex in DMSO, the labelling experiments with both SiFA-Asn(AcNH-β-Glc)-Tyr^3^-octreotate and SiFA-Asn(AcNH-β-Glc)-PEGTyr^3^-octreotate exhibited a ^18^F-incorporation of about 40% and 60% when 10 nmol and 25 nmol of precursor were used, respectively. SiFAlin was obtained by inserting a permanent positive charge on the SiFA scaffold. The overall synthesis time was 15 min. The radiochemical purities ranged from 92% to 96%. The specific activities were between 29 and 56 GBq μmol^−1^ and the radiochemical yields were 38 ± 4%. Even if the introduction of the PEG spacer and the carbohydrate in the peptide sequence could not counteract the high lipophilicity of the SiFA building block, both derivatised octreotate conjugates maintained their binding affinities to the somatostatin receptor subtype 2 (sst2) and the SiFA-Asn(AcNH-β-Glc)-PEGTyr^3^-octreotate showed a high tumour uptake in an AR42J tumour mouse model. Niedermoser *et al.* developed different SiFAlin-derivatised Tyr3-octreotate (TATE).^44^ The Tyr^3^-octreotate was synthesised and derivatised at the N-terminus by the introduction of hydrophilic auxiliaries: Fmoc–NH–PEG*n*-COOH (*n* = 1, 2, 3, 5), Fmoc–Asp(O*t*Bu)–OH, Fmoc–Asn(Ac_3_AcNH-β-Glc)–OH, and Boc_2_–Aoa–OH; using standard Fmoc solid-phase peptide synthesis protocols. After cleavage of the peptides from the resin, the crude peptides were purified using semipreparative HPLC. Then, they were incubated with an excess of the corresponding SiFA-synthon (either SiFA-aldehyde or freshly prepared SiFAlin-aldehyde) in phosphate buffer at pH 4.0 to obtain the SiFA- and SiFAlin-conjugated products. Finally, the peptides were purified using semipreparative HPLC and isolated as white solids after lyophilisation. The isotopic exchange ^18^F–fluoride radiolabelling reaction was completed within 5 min at RT with 70–90% ^18^F–fluoride incorporation. The ^18^F-labeled peptides were obtained in high RCYs of 49.8% ± 5.9% (*n* = 20) and chemical and radiochemical purities ≥98% (total synthesis time of 20–25 min). All the radiotracers displayed specific activities of 44–63 GBq mmol^−1^ using starting activities of 3.3–6.7 GBq. The modified TATE peptides showed high binding affinities to somatostatin receptor-positive tumour cells. The introduction of the novel SiFA building block SiFAlin and of hydrophilic auxiliaries demonstrated a favourable *in vivo* biodistribution profile of the novel radiotracers, resulting in high tumour-to-background ratios, although lower than those observed with the well-known ^68^Ga-DOTATATE.

## Design and strategies for ^11^C marked peptides

The ^11^C-radiolabelling of peptides for PET is common due to its presence in organic molecules, but, on the other hand, it has been poorly explored, due to its short half-life of 20 min. Even though, some peptide derivatives, *i.e.*, somatostatin and RGD derivatives, have been synthesised.

### ^11^C methylation

^11^C methylation is one of the methodologies used for peptide radiolabelling, through the direct methylation of cysteine residues. The first reported synthesis of ^11^C-labelled peptides was the labelling of homocysteine residue which yielded a radiolabelled methionine peptide. In particular, it was a reductive deprotection followed by [^11^C]methyl iodide ([^11^C]CH_3_I) methylation of pentameric enkephalin peptides containing homocysteine residues protected with a benzyl group.^45^ More recently, single-step direct-methylation of cysteine has been described by Chin *et al.* using [^11^C]methyl triflate ([^11^C]MeOTf) for the methylation of three peptides: the commercially available glutathione (GSH), the decapeptide (Trp–Tyr–Trp–Ser–Arg–Cys–Lys–Trp–Thr–Gly) and Cys(Me)–[Tyr 3-octreotate].^46^ [^11^C]MeOTf was obtained by reaction of [^11^C]CH_3_I with silver triflate in an online flow-through process at 175 °C using an N_2_ gas flow of 20 mL min^−1^. Then, to a freshly prepared solution of each peptide (1 equiv.) and aqueous NaOH (from 6 to 40 equiv.) in DMSO was bubbled freshly produced [^11^C]MeOTf in N_2_ carrier gas at 20 °C for 20 seconds without stirring. After quenching the reaction with TFA, the mixture was analysed and/or purified by RP-HPLC. Through the methylation of the decapeptide and of the Cys(Me)–[Tyr 3-octreotate], they demonstrated the regiospecificity of [^11^C]MeOTf towards cysteine residues on peptides containing various *N*-, *O*-, and *S*-nucleophilic amino acid side-chains. [^11^C]Cys(Me)–[Tyr3-octreotate] was obtained with a maximum radioactivity level of 41.8 MBq and a maximum specific activity of 96.3 MBq μmol^−1^ starting from *ca.* 340 MBq of [^11^C]MeOTf. While the radiochemical conversion of the first two radiolabelled peptides was between 75% and 65% non-decay-corrected (NDC), the one for the octreotate derivative was about 52% NDC. [^11^C]Cys(Me)–[Tyr3-octreotate] was isolated in non-decay-corrected radiochemical yields of 11 ± 2% (*n* = 3) in about 30 min. Other studies are underway to improve the efficacy of the process.

### [^11^C]CH_2_O

Henriksen *et al.* used the *p*-[^11^C]methoxy-benzaldehyde ([^11^C]MB-CHO) as a prosthetic group for labelling an aminooxyacetic acid functionalised carbohydrate analogue of Tyr3-octreotate.^47^ [^11^C]MB-CHO was synthesised by bubbling [^11^C]CH_3_I through a solution of 4-hydroxy-benzaldehyde (1.1 equiv.) and sodium methoxide (1 equiv.) in DMF followed by heating at 40 °C for 5 min. The fraction of the product was diluted with water, applied onto a Strata X cartridge and then eluted in MeCN into a vial containing 0.5–0.1 μmol peptide and 5 μl of a 2.5% solution of TFA in water. The mixture was heated at 80 °C for 7 min and subjected to HPLC. This oxime-mediated coupling gave the desired ^11^C-labelled peptide in 21 ± 5% decay-corrected yield (*n* = 4) in about 1 h. The radiochemical purity was >97%, the radiochemical yield was in the range of 55–72% and the specific activity at end of synthesis of 22–28 GBq μmol^−1^. Even if the radiotracer exhibited high and specific tumour targeting, the first-pass hepatic and intestinal accumulation was not ideal, probably due to the high lipophilicity. Moreover, this chemoselective radiolabelling leading to the formation of oximes, required time-consuming multi-step synthesis, often not suitable with the short half-life of ^11^C, and this severely reduced its application. Hanyu *et al.* developed the synthesis of [1-^11^C]1,2,3,4-tetrahydro-β-carboline-3-carboxylic acid ([1-^11^C]Tpi) (1-[^11^C]Tpi) from the corresponding Trp-containing RGD peptide by using a Pictet–Spengler reaction with [^11^C]formaldehyde.^48^ The [^11^C]CH_2_O for the radiosynthesis was prepared from [^11^C]CH_3_I as previously described.^49^ Then, [^11^C]CH_2_O/DMF solution formed was added to a reaction vial containing Cyclo[Arg–Gly–Asp–d-Tyr–Lys(Trp)] hydrochloride in water. The reaction mixture was heated at 100 °C for 5 min, after which the mixture was quenched by the addition of water and subjected to RP-HPLC. The radiochemical yield of the [1-^11^C]Tpi-containing RGD at the end of synthesis was 5.9 ± 1.9% (*n* = 4), for a total synthesis time of about 35 min. The specific activity was 85.7 ± 9.4 GBq μmol^−1^ at the EOS.

### Pd mediated aminocarboxylation

**Scheme 7:**
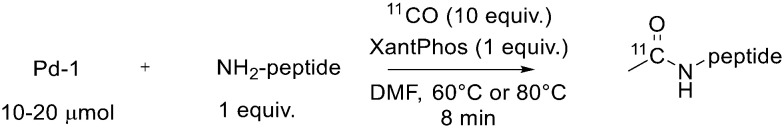
A general example of *N*-^11^C-acetylation of peptides.

Another route to obtain ^11^C labelled peptides is based on aminocarboxylation mediated by palladium employing [^11^C]CO. Andersen *et al.* reported the synthesis of *N*-[^11^C]acetylated peptides on terminal amine or lysine residues *via* Pd-mediated carbonylation using [^11^C]CO as shown in Scheme 7.^50^ The ^11^C acetylation, carried out in mild conditions, demonstrated to be N-terminal selective, even if there was a competing reaction on cysteine residues, the *S*-acetylation. In any case, the labelled molecules were obtained in acceptable radiochemical yields and with high specific activity suitable for *in vivo* studies. They carried out, for example, the ^11^C-acetylation of the cyclo-RGDfK lysine to obtain [^11^C]acetyl cRGDfK with a radiochemical conversion from ^11^CO of 63 ± 5% (*n* = 3), RCY = 37%, the specific activity of 1.48 GBq (40 mCi) and radiochemical purity = 99%.

### ^11^CN labelling

Zhao *et al.* developed a direct [^11^C]cyanide labelling of unprotected peptides carrying a cysteine residue through a palladium-mediated sequential cross-coupling reaction.^51^ In this method, the nucleophilic character of both the peptide and labelling reagent ([^11^C]HCN as [^11^C]cyanide source) was exploited in order to obtain a “nucleophile-nucleophile coupling” directly *via* Pd-complex dihaloarene oxidative addition. The mild reaction conditions and high chemoselectivity allow to chemoselectively label a cysteine in the presence of other potentially nucleophilic moieties. The key points of this strategy were that it could be successfully used on a very small amount of the peptide precursor and that the overall process time, including the purification, was only 15 min.

## Design and strategies for ^68^Ga marked peptides

**Fig. 4:**
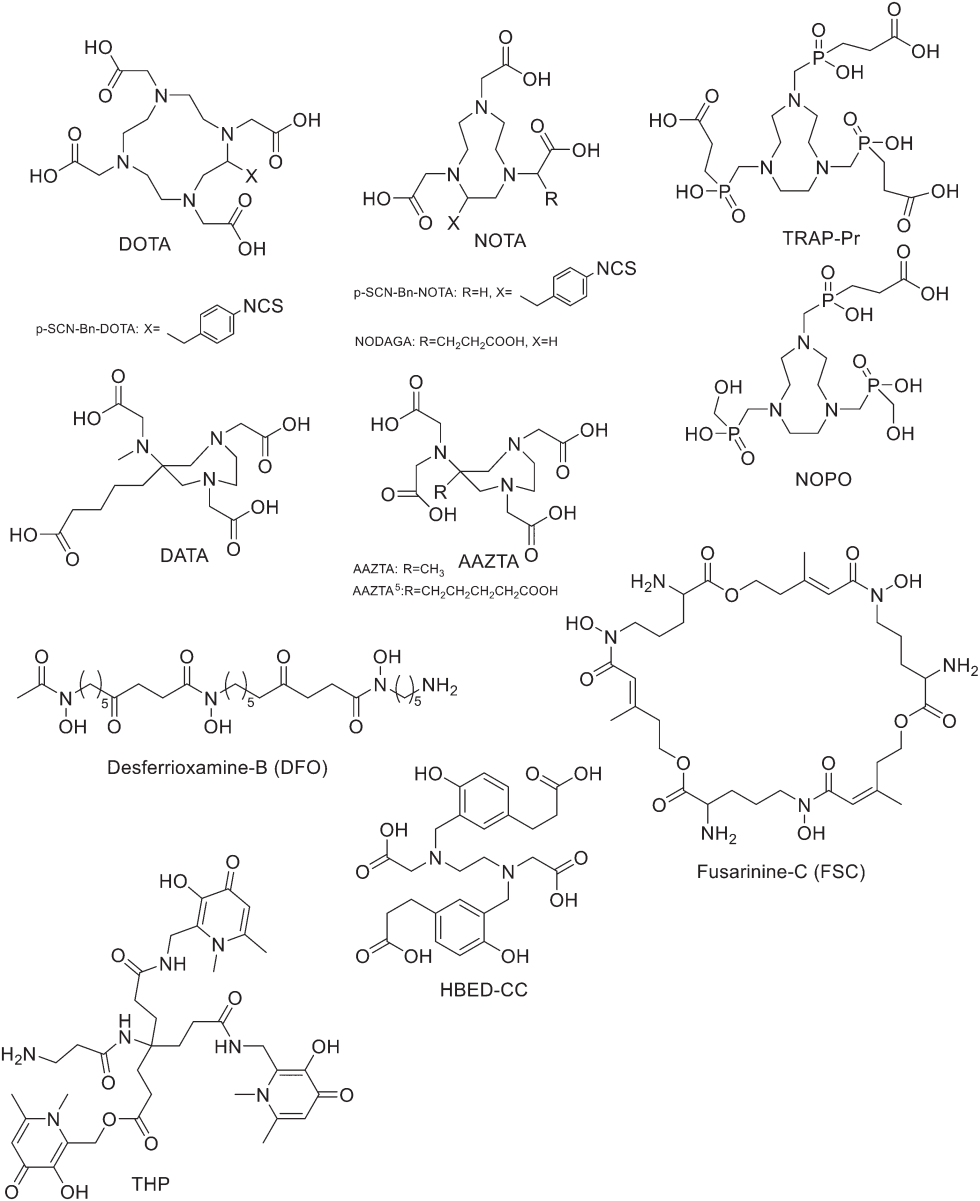
The most common BFCAs for ^68^Ga-labelled peptides.

Since ^68^Ge/^68^Ga-generators became available, ^68^Ga has been considered an attractive isotope for PET imaging. Gallium is a hard Lewis acid and generally creates octahedral complexes with hard Lewis bases, such as oxygen and nitrogen atoms, with a maximum coordination number = 6. It is also able to form four- and five-coordinated complexes.^52,53^ The most common bifunctional chelating agents (BFCAs) for ^68^Ga are macrocyclic compound like DOTA (1,4,7,10-tetraazacyclododecane-1,4,7,10-tetraacetic acid), NOTA ((1,4,7-triazanonane-1,4,7-triyl)triacetic acid) and their derivatives, and others. (Fig. 4). External substituents, such as carboxyl groups, allow conjugation to various targeting molecules.^54^

### DOTA and its derivatives

The functionalisation of peptides with DOTA can be achieved both from the ring methylene carbons^55^ and through one of the four carboxylates of the macrocycle.^56^ Concerning the second method, activation of a carboxylate group with isobutyl chloroformate^57^ allows coupling to the peptide.^58^ An inconvenience of this method is the low solubility of DOTA^58^ in dry organic solvents.^56^ The commercially available tri-protected DOTA-*tris*-(*t*-Bu ester) with a free carboxyl group is totally compatible with Fmoc-based solid-phase peptide synthesis (SPPS) but it is expensive and contains chemical impurities of both the dialkylated and tetra-alkylated cyclen. In 1999 Peterson *et al.* developed a method to avoid these inconveniences by synthesising DOTA stepwise on the peptide N-terminus, using SPPS.^59^ After the synthesis of the peptide, the last N-terminal Fmoc amino acid was acylated by reaction with bromoacetyl bromide or chloroacetyl chloride in presence of DIEA in dry NMP, followed by coupling with cyclen (solution in anhydrous DCM). Next, the free amines of cyclen were protected by reaction with *tert*-butyl bromoacetate in presence of DIEA in NMP. Cleavage of the DOTA-peptide amide from the resin followed by HPLC purification gave the desired compounds with yields from 18% to 37% depending on the peptide chain. Even if the DOTA-peptide yields were similar to those reached with other strategies, such as the activation of only one carboxylic group on the macrocycle, the time of synthesis of the DOTA peptide amide was significantly reduced; all the by-products and unreacted reagents were eluted during the wash steps. More recently, Okarvi *et al.* synthesised ^68^GaDOTA-linked bombesin (BBN) peptides based on the previous synthesis but employing bromoacetic acid (activated *in situ* with HOBt and DIC) instead of bromoacetyl bromide for N-terminus acylation, which gave efficient acylation and better yields (up to 40%).^60^^68^Ga radiolabelling of DOTA-coupled BBN peptides was conducted by mixing ∼20 μg BBN peptide with sodium acetate buffer and ^68^GaCl_3_ followed by heating until the labelling reaction was complete. For all the synthesised radiotracers, a high radiochemical purity was found, the radiolabelling efficiency ranged from 90 and 95% and the specific radioactivity was >250 Ci mmol^−1^. The critical step of solid-phase DOTA synthesis was the monoalkylation of cyclen and the only way to achieve it was to use an excess molar amount of cyclen (10–15 equiv. to resin). Next, to furnish the fully alkylated target compound, 3.0–3.3 equiv. to resin of *tert*-butyl 2-bromoacetate and an optimized reaction time (∼3 h) were employed. Moreover, thanks to this approach, the tetraalkylating product formation, which was usually observed when DOTA-coupled peptides are prepared from the commercially available DOTA-tris, was only occasionally observed. These DOTA-coupled BBN peptides prepared from cyclen by using SPPS demonstrated similar potency and efficiency to commercially available DOTA-tris for bombesin/gastrin-releasing peptide (BBN/GRP) receptor-positive tumours. Recently, the novel chelator DATA (6-amino-1,4-diazepine-triacetate) has been synthesized. Its advantage is the high yield and the fact that the radiolabelling of a DATA-based peptides, namely a octreotide derivative (TOC), took place at RT in contrast to DOTA, which needs a temperature of 95 °C for effective labelling.^61^ Sinnes *et al.* compared [^68/nat^Ga]Ga-DATA-TOC to [^68/nat^Ga]Ga-DOTA-TOC in different *in vitro* and *in vivo* studies. DATA-TOC was labelled with ^68^Ga with a radiolabelling efficiency >95% in less than 10 min at RT, achieving a molar activity of up to 35 MBq nmol^−1^. The two radiotracers displayed similar properties in terms of *in vitro* affinity and *in vitro* internalisation to SST-positive cell lines, as well in terms of organ distribution and uptake kinetics. These results highlighted the possible use of the DATA-chelator as a promising tool for ^68^Ga-radiolabelling.^62^ Recently, Nock *et al.* developed a GRPR antagonist, in which DOTA was linked to the peptide through a *p*-aminomethylaniline-diglycolic acid as linker (^68^Ga-DOTA-*p*-aminomethylaniline-diglycolic acid-d-Phe–Gln–Trp–Ala–Val–Gly–His–Leu–NHEt, known as NeoBOMB1).^63^ The RCP was >99%. The use of ^68^Ga-NeoBOMB1 PET/CT in prostate cancer patients highlighted the translational impact of this new tracer. Ferguson *et al.* proposed a ^68^Ga-labelled DOTA complexes conjugated to GRPR antagonist BBN2 using aminovaleric acid (Ava) as a linker.^64^ The metabolically stabilised bombesin peptide Ava–Gln^7^–Trp^8^–Ala^9^–Val^10^–Sar^11^–His^12^–FA01010^13^–Tle^14^–NH_2_ (Ava-BBN2) was linked to pSCN-Bn-DOTA (1.3 equiv.) in presence of TEA in DMF. The mixture was incubated at 37 °C for 16 h and then the peptide was cleaved from the resin. After preparative HPLC purification and lyophilisation, DOTA-Ava-BBN2 was obtained as a white solid in 85% isolated yield. Peptide DOTA-Ava-BBN2 (50 μg) was then radiolabelled with ^68^Ga and at pH 4.5 using 4 M NaOAc buffer (pH 9.3) at 95 °C for 20 min. After solid-phase extraction (SPE) purification, ^68^Ga-DOTA-Ava-BBN2 was obtained in 70% decay corrected (DC) radiochemical yield at radiochemical purity >97%. The effective molar activity was in the range of 3–8 GBq μmol^−1^ using starting activity of 100 MBq for ^68^Ga. Fast clearance of radioactivity from the blood and non-target tissues was observed due to the small size and hydrophilicity of the radiotracer. This resulted in favourable image contrast and high tumour-to-muscle ratios. Thus, the study confirmed the good GRPR-targeting tools of metabolically stabilised bombesin antagonists like BBN2 peptides for PET imaging of prostate cancer. Fanelli *et al.* presented a series of bioconjugates constituted by stabilised NT[8–13] analogues radiolabelled with ^68^Ga using DOTA as a chelator.^65^ The fragment NT[8–13] represents the shortest active sequence of neurotensin (NT), an endogenous peptide which shows high affinity and specificity to neurotensin receptors NTS_1_ and NTS_2_. Radiolabelled neurotensin tracers have low plasma stability and therefore suffer from inadequate bioavailability for high uptake in tumours. In their study it is reported the development of ^68^Ga-radiolabelled neurotensin analogues with better radiopharmaceutical characteristics thanks to the introduction of the silicon-containing amino acid trimethylsilylalanine (TMSAla). All the obtained NT analogues were attached to the protected chelator DOTA(*t*Bu)_3_–OH *via* the amino piperidin-1-yl-acetic acid ((4)-APAc) linked to the N-terminal amine of the peptide fragment. Depending on the peptide chain, the synthesis was carried out either *via* SPPS or in solution. In the first case the last amino acid, after removal of Fmoc, was reacted with Fmoc–4-APAc–OH in presence of HATU (4 equiv.) and DIPEA (10 equiv.) in DMF. After each deprotection cycle, the solvent was removed by filtration and the resin was washed with DMF. DOTA(*t*Bu)_3_–OH was added together with HATU (4 equiv.) and DIPEA (10 equiv.) in DMF. For the conjugation of Fmoc–4-APAc–OH and DOTA-(*t*Bu)_3_–OH, the reaction time was from 2 h to 5 h. After cleavage from the resin and final deprotection, the crude compounds were injected into preparative HPLC and the final bioconjugates were obtained with yields ranging from 58% to 81%. All analogues were radiolabelled with ^68^Ga with 60% yield, high radiochemical purity (>95% after purification) and high apparent molar activity of 12.5 ± 1.3 GBq μmol^−1^. The peptide sequence where the Arg^8^–Arg^9^ pair was replaced with Lys^8^–Lys^9^ and TMSAla was inserted in position 13, showed to be the most promising one in terms of binding affinity toward NTS_1_ and good selectivity over NTS_2_. It also showed improved stability in human plasma with respect to the reference compound and a very good specific tumour uptake in mice bearing HT-29 tumours. Loktev *et al.* used DOTA to label a fibroblast activation protein (FAP)-specific enzyme inhibitor (FAPI) with ^68^Ga.^66^ DOTA chelator was linked to FAPI through piperazine as a linker which was linked to the FAPI scaffold by using 1-bromo-3-chloropropane as a spacer. The amination was conducted with 1-tertbutoxycarbonylpiperazine in the presence of KI as a catalyst. The Boc-piperazine derivative of FAPI (1 equiv.), after Boc removal by using 4-methylbenzenesulfonic acid, was reacted with DOTA-*p*-nitrophenol ester (1.5 equiv.) and TEA in DMF for 2 h. Then, the reaction mixture was diluted with water and purified by HPLC and after lyophilisation, the final compound was obtained (68% yield). The radiolabelling with ^68^Ga was conducted at 95 °C for 10 min. Radiolabelled FAPI showed high specificity, affinity, and rapid internalisation into FAP-expressing cells *in vitro* and *in vivo*. High intratumoral uptake and fast body clearance were demonstrated by the biodistribution studies on mice bearing FAP-expressing (HT-1080-FAP) as well as FAP-negative (Capan-2) cancer cell lines. All these properties resulted in fast and high-contrast images. Very recently, Duan *et al.*^67^ reported a series of PSMA (prostate specific membrane antigen) inhibitors based on the oxalyldiaminopropionic acid-urea (ODAP-urea) scaffold, which is one of the few examples modifying the glutamate-like moiety that binds to the PSMA's S1' sub-binding domain with an affinity that is comparable to or even greater than the Glu-ureas.^68^ In particular, twelve ODAP-urea-based ligands were synthesised and radiolabelled with ^68^Ga using DOTA as chelator. A variety of linkers have been used between ODAP-urea and DOTA; two groups of compounds were synthesised with the targeting moiety (ODAP-urea-Lys) either conjugated to 4-alkyloxybenzoic acids or to aromatic amino acids. All the ligands were synthesised *via* solid-phase synthesis which was performed according to Fmoc peptide synthesis protocol. The synthesis cycle consisted of: i) Fmoc cleavage: 20% piperidine in DMF, ii) DMF washings, iii) coupling, and iv) DMF washings. The resin was treated with 2 mL DCM for 5 min three times and 2 mL DMF for 5 min three times before used. The resin was washed thoroughly before it was incubated with HBTU-preactivated amino acid, amino acid derivatives or tris–*t*Bu-DOTA. DDE was cleaved by 2% hydrazine hydrate/DMF for 3 min twice. Cleavage from the solid support was performed with TFA/water/triisopropylsilane (95/2.5/2.5, vol/vol/vol) over 3 h at RT. All ligands were obtained with moderate yield (12.1–44.4%) and high purity (>95%) after HPLC purification. The preparation of ^68^Ga-labeled compounds was carried out by incubating 30 μg of each ligand with a mixture of 1.0 mL ^68^GaCl_3_ (0.05 N HCl) eluent with radioactivity of 185–370 MBq and 65 μL sodium acetate (1 mol L^−1^) at 85–90 °C for 10 min. The product was purified by a Sep-Pak® Light C18 cartridge and the ^68^Ga-labeled compound was obtained by eluting the cartridge with 0.6 mL 80% EtOH. The radiochemical purity of ^68^Ga-labelled compounds was analysed by radio-HPLC and resulted >98% with radiochemical yields >90%. Ligands with aromatic acids conjugated to ODAP-urea-Lys demonstrated higher affinity to PSMA than did those with alkoxybenzoic acid and, of this, one compound showed to be the most promising one with higher uptake than [^68^Ga]Ga-PSMA-617 in tumour-bearing mice at 6.43 ± 0.98% IA/g *vs.* 3.41 ± 1.31% IA/g at 60 min post-injection. In human studies, the normal organ biodistribution of this lead ligand was similar to that of [^68^Ga]Ga-PSMA-617 except for within the urinary tract, in which the new compound demonstrated lower uptake.

### NOTA and its derivatives

**Fig. 5:**
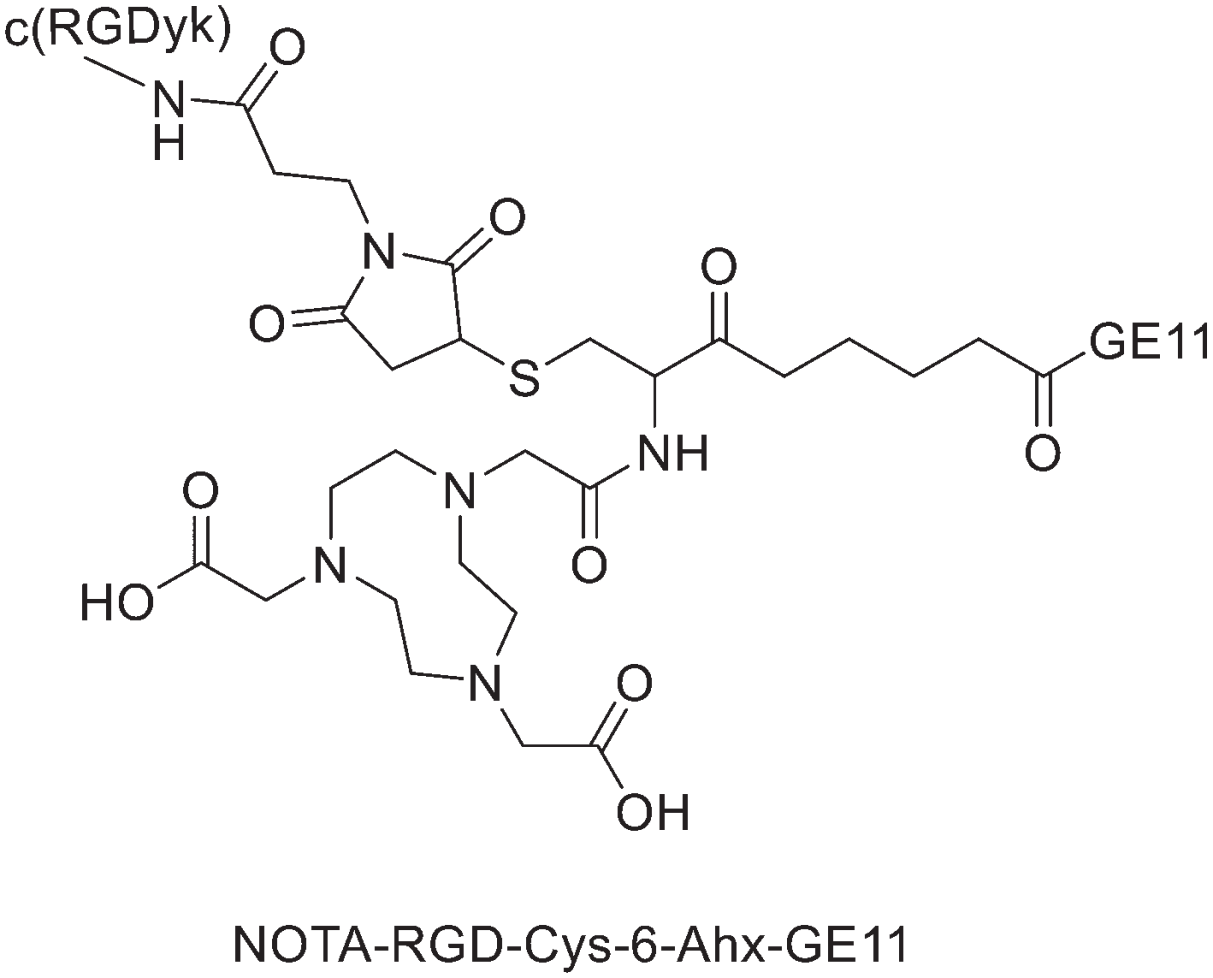
Chemical structure of NOTA-RGD-Cys-6-Ahx-GE11.

It has been found that the use of NOTA and its derivatives (Fig. 4), due to their nine-membered ring structure, increases the complex stability and allows complexation under milder conditions compared to DOTA. This has been proven, for example, by Kumar *et al.* who estimated the properties of [^68^Ga] NODAGA and DOTA-peptide conjugate targeting VPAC [combined for vasoactive intestinal peptide (VIP) and pituitary adenylate cyclase-activating peptide (PACAP)] receptors on tumour cells.^69^ For the radiolabelling with ^68^Ga, 20 μg of peptide conjugate in 200 μl of deionized water and 4.5 ± 0.5 mCi (20 μl) of [^68^Ga]GaCl_3_ were incubated at 90 °C for 30 min and pH was adjusted to 7.2 ± 0.2 with 0.1 M NaOH. The radiochemical purities were 96.3 ± 0.5% for [^68^Ga]NODAGA-peptide and 92.7 ± 5.8% for [^68^Ga]DOTA-peptide. The specific activity of [^68^Ga]NODAGA/DOTA-peptide each, based on the availability of [^68^Ga]GaCl_3_, varied from 37.2 ± 2.1 to 49.2 ± 2.3 GBq μmol^−1^. They found that NODAGA was better than DOTA in terms of radiolabelling and kinetics and, although both agents displayed relatively low *in vivo* stability, [^68^Ga]NODAGA-peptide showed better stability and greater affinity for VPAC-expressing BT474 breast cancer cells than [^68^Ga]DOTA-peptide. Indeed, breast cancer tumours were delineated with [^68^Ga]NODAGA-peptide but not with [^68^Ga]DOTA-peptide after PET/CT imaging studies. Li *et al.* developed the new radiotracer ^68^Ga-NOTA-MAL-Cys^39^-exendin-4 that specifically targets the glucagon-like peptide-1 receptor (GLP-1R).^70^ Lately, a C-terminal or N-terminal cysteine has been attached to exendin-4 analogues to allow site-specific labelling with a maleimide-selective prosthetic reagent. In this case, NOTA-MAL-Cys^39^-exendin-4 was obtained by the reaction of NOTA-maleimide (NOTA-MAL) (1.2 equiv.) with Cys^39^-exendin-4 in 0.2 M ammonium acetate. Then, MeCN was added, and the mixture was stirred at 40 °C overnight. After semi-preparative HPLC purification and lyophilisation, the desired product was obtained in 70% yield. Then, [^68^Ga]GaCl_3_ was eluted from a ^68^Ge/^68^Ga-generator with 0.05 M HCl, and NOTA-MAL-Cys^39^-exendin-4 was radiolabelled with heating at 100 °C for 10 min (RCY = 91.6 ± 2.8%). The radiochemical purity was higher than 95% while the specific activity was at least 8.5 GBq μmol^−1^. Their results showed that ^68^Ga-NOTA-MAL-Cys^39^-exendin-4, which was rapidly accumulated in the tumour and cleared quickly *in vivo*, could specifically target GLP-1R and gave good image contrast. However, the uptake of radioactivity in the kidneys was much higher than in other organs; high radioactivity in the kidneys means that the tracer will mainly have renal clearance, but this high absorption can lead to radiation-induced kidney damage. This could pose a threat to the potential use of this radiotracer. RGD (as mentioned before) and GE11 peptides specifically target integrin αVβ3 and EGFR, respectively. Recently, Yu *et al.* synthesised a NOTA-RGD-GE11 heterodimer labelled with ^68^Ga.^71^ The NOTA-RGD-GE11 heterodimeric peptide was synthesised in three steps: (1) NOTA-Cys-6-Ahx-GE11 synthesis, (2) maleimidopropyl-c(RGDyK) synthesis, and (3) NOTA-Cys-6-AhxGE11 and maleimidopropyl-c(RGDyK) conjugation with a thioether linkage (Fig. 5).

**Scheme 8:**
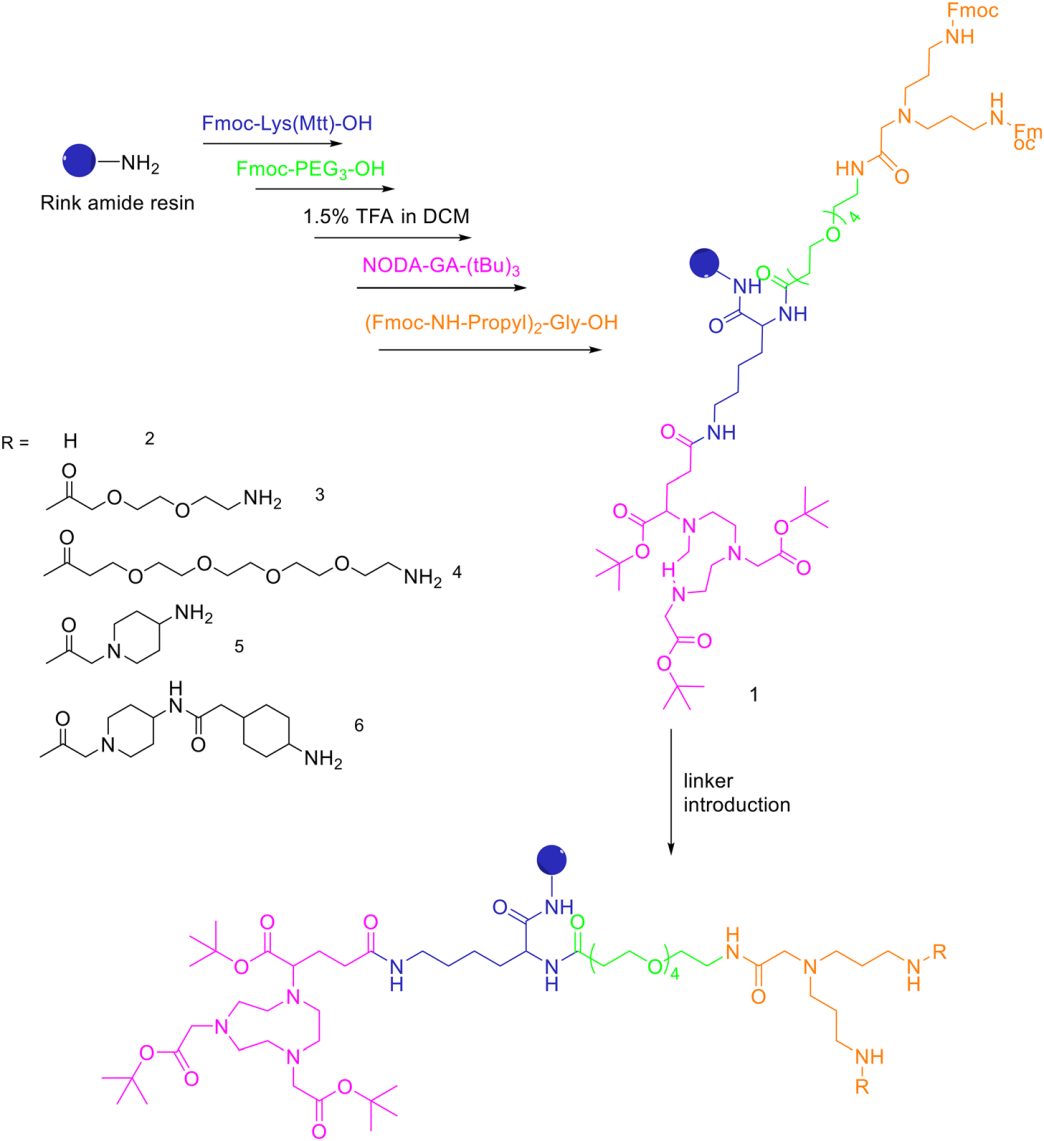
Synthesis of the protected, chelator-modified intermediate 1 and of the bis-amines 2–6 obtained by modification of 1 with different linkers.

The cysteine in the NOTA-Cys-6-Ahx-GE11 peptide served as a link furnishing both an amino group for the NOTA coupling and a thiol group for the maleimidopropyl-c(RGDyK) conjugation. The 6-aminohexanoic acid divided the two peptides. For NOTA-Cys-6-Ahx-GE11 synthesis, the last amino acid was Fmoc deprotected and reacted with Fmoc-aminohexanoic acid in presence of HBTU, HOBt, and DIEA (2/2/10) in DMF. Then, after Fmoc removal, the amino group of the Ahx acid was coupled with Fmoc–Trt–cysteine in presence of HBTU, HOBt, and DIEA (2/2/5) by microwave-assisted coupling. After Fmoc deprotection, it was reacted with NOTA(*t*Bu)_2_ in presence of HBTU, HOBt, and DIEA (2/2/10) in DMF by microwave-assisted coupling. Maleimidopropyl-c(RGDyK) was obtained by reacting c(RGDyK) (1 equiv.) with 3-maleimidopropionic acid *N*-hydroxysuccinimide ester (BMPS; 1.1 equiv.) in DMF and TEA under inert atmosphere at RT for 6 h. The crude product was purified by RP-MPLC and yielded maleimidopropyl-c(RGDyK) (32%). Then, for NOTA-Cys-6-Ahx-GE11 and maleimidopropyl-c(RGDyK) coupling, a mixture of NOTA-Cys-6-Ahx-GE11 (1 equiv.) and maleimidopropyl-c(RGDyK) (1 equiv.) in MeCN and phosphate buffer (pH 6.0) was reacted by rotation and vibration at RT for 12 h. The product was purified by RP-HPLC and obtained in 43% yield. NOTA-RGD-GE11 radiolabelling was conducted by eluting ^68^Ga from a ^68^Ge/^68^Ga generator with 0.1 M HCl and applying it directly to the reaction. The mixture was heated at 90 °C for 15 min. The overall synthesis time was 40–45 min, from the ^68^Ga elution to the product reconstitution, with an 85% decay corrected yield, a radiochemical purity >98%, and specific activity reaching 44.3 MBq nmol^−1^. The labelling yield was >90% in these conditions against the yield of 70% for the reaction conducted at RT. More recently, Chen *et al.* investigated the properties of ^68^Ga-NOTA-RGD-GE11 for dual receptor imaging.^72^ In particular, the *in vitro* and *in vivo* properties of [^68^Ga]Ga-NOTA-RGD-GE11 were evaluated and compared with those of monomeric peptides [^68^Ga]Ga-NOTA-RGD and [^68^Ga]Ga-NOTA-GE11. [^68^Ga]Ga-NOTA-RGD-GE11 demonstrated higher tumour uptake than [^68^Ga]Ga-NOTA-RGD and [^68^Ga]Ga-NOTA-GE11 in biodistribution and PET/CT imaging studies. Radiolabelled heterobivalent peptidic ligands (HBPLs) represent tumour imaging agents which can offer many advantages compared to monovalent peptide receptor ligands, due to their ability to target different receptors. Lindner *et al.* designed and synthesised different HBPLs, including a GRPR-binding (BBN 7–14) and a VPAC1R-targeting (PACAP-27) peptide labelled with ^68^Ga.^73^ Functionalised branched moieties employed as scaffolds for peptide heterodimerization were obtained following the procedures described in Scheme 8.

A standard rink amide resin was used to build the scaffold by following coupling of Fmoc–Lys(Mtt)–OH, Fmoc–PEG_3_–OH, followed by lysine side chain deprotection using diluted TFA, conjugation of NODA-GA-(*t*Bu)_3_ in this position and subsequent conjugation of the branching amino acid *N*,*N*-bis(*N*′-Fmoc-3-aminopropyl)-glycine ((Fmoc-NH-propyl)_2_-Gly-OH), yielding the branched intermediate 1. The Mtt-protecting group of the lysine was cleaved using 1.75% TFA in DCM for 35 min and NODA-GA-(*t*Bu)_3_ was coupled to lysine using PyBOP rather than HBTU as coupling reagent and extended reaction times of 2 h. Afterwards, *N*,*N*-bis(*N*′-Fmoc-3-aminopropyl)-glycine potassium hemisulfate was coupled, followed by Fmoc–PEG_1_–OH, Fmoc–PEG_3_–OH or one or two copies of Fmoc-ACMP (Fmoc-4-amino-1-carboxymethylpiperidine). The resulting NODA-GA-bis-amino-dendrons (2–6) were cleaved from the resin using a mixture of TFA/TIS/H_2_O of 80/10/10 (v/v) for 3 h and injected into semipreparative HPLC after evaporation of the volatile materials. The products were isolated and lyophilised with overall yields from 31% to 64%. The bis-amines 2–6 were further reacted in solution with succinimidyl *p*-formyl benzoate to obtain the respective bis-aldehydes, which acted as the final scaffolds for the click chemistry-based heterodimerization of the aminooxy-modified peptides. ^68^Ga-Radiolabelling of NODAGA-modified peptide heterodimers and peptide monomers (10 min at 40–45 °C) gave the radiolabelled products with RCP ranging from 95% to 99% and non-optimized molar activities of 25–61 GBq μmol^−1^ starting from 246–612 MBq of ^68^Ga^3+^. They found that the heterobivalent analogues displayed similar uptake into the tumour cells to those of the respective monomers, indicating that both peptides are still able to reach their target receptors. Furthermore, they found that in cases of overall low receptor densities, heterobivalent peptides showed higher tumour cell uptake than peptide monomers.

### TRAP and its derivatives

To overcome the disadvantage of NOTA-like chelators, namely the slow formation of Ga(iii) complexes, the exchange of the carboxylate moieties by phosphinate ones proved to be a promising approach. TACN-*N*,*N*′,*N*′′-tris-(methylenephosphinic acid) chelators (TRAP, Fig. 4) demonstrated much faster Ga^3+^ complexation kinetics. Triazacyclononane-phosphinic acid is a nine membered ring which contains phosphinic acid groups. TRAP chelators form very stable complexes with Ga^3+^ over a large pH range and at RT, and the synthesis of homo- and heteromultimeric bioconjugates can be done straightforwardly. Notni *et al.* developed a TRAP-based cyclo(RGDfK) trimer with high integrin affinity.^74^ The ^68^Ga^3+^ complexation performance was compared for TRAP ligands, DOTA, and NOTA. They found that ^68^Ga was incorporated nearly quantitatively (>95%) at much lower ligand concentrations by the TRAP ligands and, given the low p*K*_a_ of phosphinic acid moieties (0.7–1.5), also in a strongly acidic environment. At first, TRAP-Pr (Fig. 4, 1 equiv.) was derivatised with the linker H_2_N-dPEG™(4)-COO*t*Bu (PEG_4_, 5 equiv.) in presence of HATU (8 equiv.) and DIPEA (10 equiv.) in DMSO for 10 min. After diafiltration, and acidic deprotection, purification by preparative HPLC afforded the final compound (77%). Then, TRAP-PEG_4_ (1 equiv.) was reacted with the protected cyclic pentapeptide, cyclo(RGDfK)(Pbf, *t*Bu) (4.4 equiv.), in the presence of HATU (7 equiv.) and DIPEA (16 equiv.) in DMSO for 10 min. After precipitation and acidic deprotection, the crude product was purified by preparative HPLC and the desired cyclo(RGDfK) trimer was obtained in 50% yield. These researchers also synthesised the trimer using click chemistry (CuAAC). After derivatization of TRAP-Pr with propargylamine and the coupling between cyclo(RGDfK)(Pbf, *t*Bu) and 5-azidopentanoic acid to obtain azido–cyclo(RGDfK)(*t*Bu, Pbf), TRAP-N_3_(RGD)_3_ was obtained by using Cu(OAc)_2_·H_2_O. This method of conjugation had no significant advantage over amide coupling as the yields were lower and Cu(ii) decomplexation must be performed as an additional step. ^68^Ga labelling was performed on a fully automated system without pre-purification or concentration of the generator eluate before labelling. The vial was heated to 95 °C for 5 min, then the mixture was passed through a SPE cartridge. ^68^Ga-TRAP(RGD)_3_ was obtained in high yields and with high specific activity (>4 TBq mmol^−1^) and radiochemical purities ≥99%. Even if the labelling efficiency was better at higher temperatures, TRAP-Pr was able to rapidly incorporate ^68^Ga at 25 °C and cL = 3 μm, while neither DOTA nor NOTA can be labelled under these conditions. 1,4,7-Triazacyclononane-1,4-bis[methylene(hydroxymethyl)-phosphinic acid]-7-[methylene(2-carboxyethyl) phosphinic acid] (NOPO, Fig. 4) represents an alternative to TRAP when only monomeric derivatives are required. NOPO, which has one pendant arm with a carboxylic acid moiety, and two hydroxymethyl-end-capped pendant arms, was developed by Šimeček *et al.*^75^ They conjugated cyclo(RGDfK) and NaI-octreotide (“NOC”, targeting of sst2 and 5) to the carboxylic acid of NOPO using HATU as coupling agent and DIPEA as a base (yields ranging between 40% and 51%). The intramolecular phosphinic acid ester (phosphilactone), obtained during amide coupling on the carboxylic acid moiety, was cleaved during ^68^Ga labelling, but it could also be hydrolysed under mildly acidic conditions. ^68^Ga-Labelling of NOPO was virtually quantitative at RT between pH 3 and 4 (30 μM) and at 95 °C over the range of pH 0.5–7 (10 μM) at very low ligand concentrations as classic for TRAP-type chelators. A more recent study of the same research group reported preliminary *in vivo* imaging of the somatostatin-receptor status using ^68^Ga-NOPO-NOC, which displayed high tumour and low organ uptake and clear tumour visualization and a very low background.^76^

### AAZTA and its derivatives

Another family of bifunctional chelators is 1,4-bis(carboxymethyl)-6-[bis(carboxymethyl)]amino-6-methylperhydro-1,4-diazepine (AAZTA, Fig. 4) and its derivatives. The AAZTA chelators have the capability to form complexes with trivalent radiometals under mild conditions. AAZTA was at first designed for the coordination of Gd^3+^ to be employed as a contrast agent in MRI. The synthesis of AAZTA was firstly reported in 2004 by Aime *et al.* and consisted in the preparation of the heptadentate macrocycle through a double nitro-Mannich cyclocondensation.^77^ In the last years, the original AAZTA structure has been modified to obtain several AAZTA analogues to be used as chelating agents. Sinnes *et al.* synthesised the bifunctional derivative of AAZTA: AAZTA^5^, which bears a pentanoic acid moiety (Fig. 4), and conjugated it to PSMA-617 (prostate specific membrane antigen), then labelled the construct with ^68^Ga.^78^ AAZTA^5^(*t*Bu)_4_ was synthesised in five steps^79^ and conjugated to the PSMA-617 backbone on solid phase by using standard amide coupling. AAZTA^5^(*t*Bu)_4_ (1 equiv.) was mixed with HATU (1 equiv.), HOBt (2.8 equiv.) and DIPEA (2.8 equiv.) in dry DMF (2 mL) and shaken for 20 min. The solution was added to PSMA-617 (resin) (0.7 equiv.), soaked in dry DMF and shaken overnight at RT. Finally, after the cleavage from the resin with TFA, the solution was concentrated and purified *via* HPLC and a white solid was obtained (12% yield). Radiolabelled AAZTA^5^-PSMA-617 conjugate demonstrated to label ^68^Ga under mild conditions in less than 5 min at RT with almost quantitative yields of >99%.

### Siderophores

Recently, also siderophores have been introduced for complexation of ^68^Ga. These compounds are low molecular weight chelators displaying very high affinity for ferric ions; plants, bacteria and fungi use them for iron acquisition and storage. Since ^68^Ga shows almost identical coordination chemistry to Fe^3+^, it can be used to radiolabel the relative siderophore. One of the first siderophores employed for labelling with ^67/68^Ga was the acyclic hexadentate tris(hydroxamate) siderophore desferrioxamine-B (DFO). Another siderophore-derivative is the macrocyclic chelator with hydroxamate groups fusarinine C (FSC) (Fig. 4). Recently, Kaeopookum *et al.* prepared mono-, di-, and trimeric c(RGDfK) analogues by reaction with FSC using click chemistry based on Cu(i)-catalysed azide–alkyne cycloaddition and then labelled with ^68^Ga.^80^ Multimeric cyclic RGD peptides were generally synthesised by two main strategies. The first one consisted in the synthesis of the cyclic RGD multimers which, subsequently, were linked to the reactive site of BFCAs such as DOTA, NOTA, and NODAGA.^81^ This method required multi-step synthesis and purification and large amounts of peptide. The second approach, as the one in this study, used a chelating agent with multiple conjugation sites as a scaffold for the multimeric radiotracer, where one RGD moiety for each site was linked. The result was a straightforward synthesis route. The acetylation of one or two amino groups of [Fe]FSC was employed to reduce the conjugation sites from three to two or one, giving acetyl[Fe]FSC ([Fe]MAFC) or diacetyl[Fe]FSC ([Fe]DAFC). The coupling reagents HOAt (4 equiv.) and HATU (4 equiv.) were reacted with 4-pentynoic acid (3 equiv.) in DMF. The mixture with 1.5, 3, or 4.5 equiv. of 4-pentynoic acid was mixed with 1 equiv. of [Fe]DAFC, [Fe]MAFC, or [Fe]FSC, respectively. DIPEA was added to reach a pH of 10–11, and the mixture was stirred at RT for 1 h. The solvent was evaporated and the crude product was purified by preparative RP-HPLC and lyophilised to obtain pentynoyl[Fe]DAFC ([Fe]DAFC(alkyn)), dipentynoyl[Fe]MAFC ([Fe]MAFC(alkyn)2), or tripentynoyl[Fe]FSC ([Fe]FSC(alkyn)3), (64%, 58% and 57% yield, respectively). Then, to cyclo(–Arg(Pbf)–Gly–Asp(O*t*Bu)–d-Phe–Lys–) with the free amino group at the lysine (1 equiv.), a mixture of HOAt (2 equiv.), HATU (2 equiv.), and 5-azidopentanoic acid (1.5 equiv.) in DMF were added. DIPEA was added to reach pH 10–11 and the mixture was stirred at RT for 30 min. After evaporation of the solvent, the residue was subjected to preparative RP-HPLC (65% yield). After removal of side protecting groups and purification by preparative RP-HPLC, (c(RGDfK)N_3_) was obtained (69% yield). 1 equiv. of [Fe]DAFC(alkyn), [Fe]MAFC(alkyn)2, or [Fe]FSC(alkyn)3 in DMF, was mixed with 2, 4, or 6 equiv. of c(RGDfK)N_3_, respectively. A freshly prepared mixture of THPTA (10 equiv.) and CuSO_4_·5H_2_O (4 equiv.) in H_2_O was added to the DMF mixture followed by sodium ascorbate (200 equiv.) solution. The mixture was stirred at RT for 2 h, and then 100 equiv. of Na_2_EDTA solution (50 mM, pH 4) was added to remove iron and the reaction mixture was stirred for 18 h. After that, the solvent was evaporated, and the crude products were purified by preparative RP-HPLC to obtain DAFC(c(RGDfK)aza): 37% yield; MAFC(c(RGDfK)aza)2: 30% yield, FSC(c(RGDfK)aza)3: 31% yield. The radiolabelling took place at RT for 10 min. [^68^Ga]GaDAFC(c(RGDfK)aza), [^68^Ga]GaMAFC(c(RGDfK)aza)2 and [^68^Ga]GaFSC(c(RGDfK)aza)3 were obtained in quantitative radiochemical yields and RCPs >99%. ^68^Ga conjugates were synthesised by replacing [^68^Ga]GaCl_3_ with 100 fold molar excess of GaBr_3_ in 200 μL of 0.1 M HCl. Of the radiotracers, [^68^Ga]GaMAFC(c(RGDfK)aza)2 showed interesting results for angiogenesis imaging.

### HBED

Another complexing agent for ^68^Ga is *N*,*N*′-bis(2-hydroxybenzyl)-1,2-ethylenediamine-*N*,*N*′-diacetic acid (HBED), which was derivatised for the alkylation of free amino groups of peptides through a 4-bromoacetamidobenzyl at the 1 position of HBED.^82^ Another strategy is based on the use of HBED-CI, where the 4-benzyl position was activated *via* isothiocyanate.^83^ HBED-CI showed to be unstable and with difficult purification; for this reason, Eder *et al.* tried to replace this compound and evaluated the properties of the tetrafluorophenolate of *N*,*N*′-bis[2-hydroxy-5-(carboxyethyl)benzyl]ethylenediamine-*N*,*N*′-diacetic acid (HBED-CC, Fig. 4), which is able to complex ^68^Ga much faster than DOTA at RT. More recently, they proposed the ^68^Ga labelled PSMA inhibitor Glu–NH–CO–NH–Lys(Ahx)-HBED-CC and compared it to the corresponding DOTA conjugate.^84^ The coupling between the free lysine of Glu–NH–CO–NH–Lys and the aminohexanoic moiety was conducted by using 2 equiv. of the Fmoc-protected 6-aminohexanoic acid, 1.96 equiv. of HBTU, and 2 equiv. of DIPEA in DMF. After cleavage from the resin by reacting with 4 mL of a 30% HFIP in DCM for 2 h at RT, the resulting *tert*-butyl protected crude product was purified *via* RP-HPLC. To conjugate HBED-CC, the purified product was reacted with an equimolar amount of HBED-CC-tetrafluorophenyl ester synthesised according to literature^85^ in the presence of 2 equiv. of DIPEA in DMF. After HPLC purification, the remaining *tert*-butyl groups were cleaved at RT in 1 h using TFA to obtain the final compound in 35% yield after purification by HPLC. Radiolabelling with ^68^Ga was conducted at pH 4.2 by incubating the conjugates in a mixture of 50–100 MBq [^68^Ga]Ga^3+^ and HEPES at RT for 2 min (radiochemical yield >99% and specific activity ranging between 500–1000 GBq μmol^−1^). The HBED-CC analogue displayed less unspecific binding and higher specific internalisation in LNCaP cells (metastatic lesion of human prostatic adenocarcinoma, ATCC CRL-1740) with respect to DOTA-conjugate. However, HBED-CC, as well as HBED, forms different isomers when complexed to Ga^3+^. For this reason, heating is employed to allow the formation of the most thermodynamically stable isomer. In a standard labelling protocol, the radiolabelling is generally performed at 95 °C or 100 °C and results in an increased formation of the thermodynamically more stable isomer of Ga-HBED-CC. If the reaction is conducted at RT, an amount of about 50% of another diastereomer is formed. In a more recent paper, Eder *et al.* studied the potential impact that the configuration of Ga-HBED-CC might have on the cell binding characteristics of the PSMA-targeted radiotracer [^68^Ga]GaPSMA-HBED-CC. Moreover, they compared its radiolabelling properties to the ones of the NOTA-labelled analogue and found that in comparison to NOTA, HBED-CC was able to complex [^68^Ga]Ga^3+^ more efficiently at low temperatures and concentrations (RT in less than 1 min). But, in these conditions, HBED-CC formed three NMR-identifiable diastereomers which could have an impact on the PSMA binding properties and on the quality of the PET/CT images. Therefore, the cell binding characteristics of [^68^Ga]Ga-PSMA-HBED-CC labelled at RT and 95 °C, respectively, were investigated in cell-based assays. Their research showed that the presence of an amount of ∼50% of the thermodynamically less favoured diastereomer did not have any negative impact on the PSMA-binding characteristics. In the case of small amounts of other diastereomers, the [^68^Ga]Ga-PSMA-HBED-CC biological functionality was not influenced. This discovery was important as a small amount of one of the thermodynamically less favoured diastereomers was still present in the labelling reaction even at 95 °C.^86^

### THPs

**Scheme 9:**
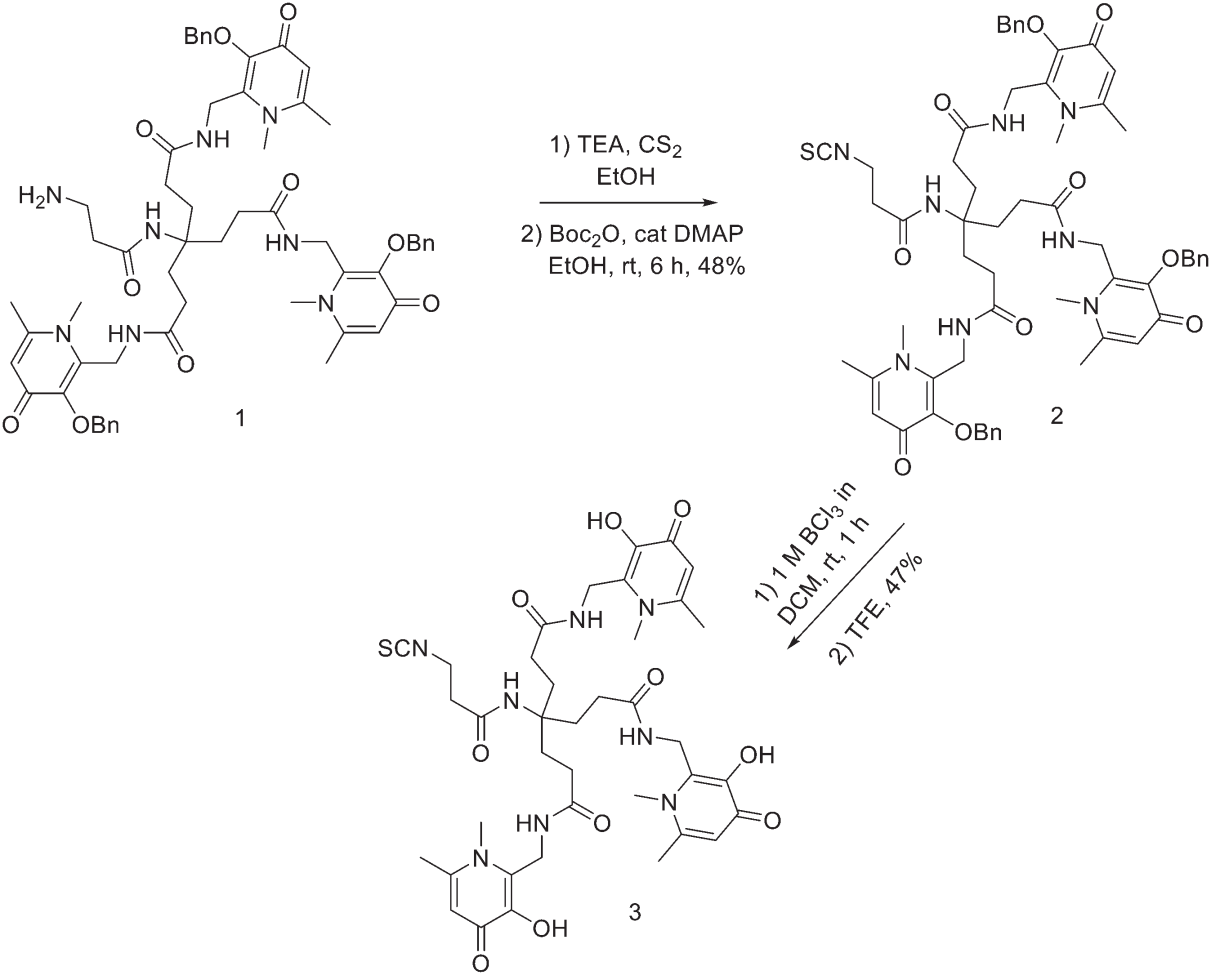
Synthesis of Tris(hydroxpyridinone) bifunctional chelator containing isothiocyanate group H_3_THP-NCS.

THPs: hexadentate tris(3,4-hydroxypyridinone) ligands (Fig. 4), show high affinities for trivalent metal ions, in fact, they are able to complex Fe^3+^ very efficiently and, in the past few years, their use has been directed towards complexation of ^68^Ga.^87^ THP-peptide conjugates are able to rapidly and quantitatively complex ^68^Ga at RT, neutral pH, and micromolar ligand concentrations. For this reason, they represent interesting compounds for kit-based radiosynthesis of ^68^Ga PET radiopharmaceuticals. Ma *et al.* worked on the synthesis of two new tris(hydroxypyridinone) chelators based on 1,6-dimethyl-3-hydroxypyridin-4-one groups that contain pendant isothiocyanates for conjugation to primary amines, to use them for labelling peptide conjugates with ^68^Ga for PET imaging.^88^ To synthesise H_3_THP-NCS (Scheme 9), the protected pyridinone hexadentate ligand containing a free amino group 1 (1.1 equiv.)^89^ was reacted with TEA (1 equiv.) and carbon disulfide (12 equiv.) in EtOH, yielding a dithiocarbamate precipitate after addition of water. It was resuspended in a solution of carbon disulfide/EtOH and di-*tert*-butyl dicarbonate (4 equiv.) and a catalytic amount of 4-dimethylaminopyridine was added, resulting in the formation of 2 (48% yield). The removal of the benzyl groups with BCl_3_ in DCM, followed by the addition of trifluoroethanol gave the desired H_3_THP-NCS 3 (47% yield).

**Scheme 10:**
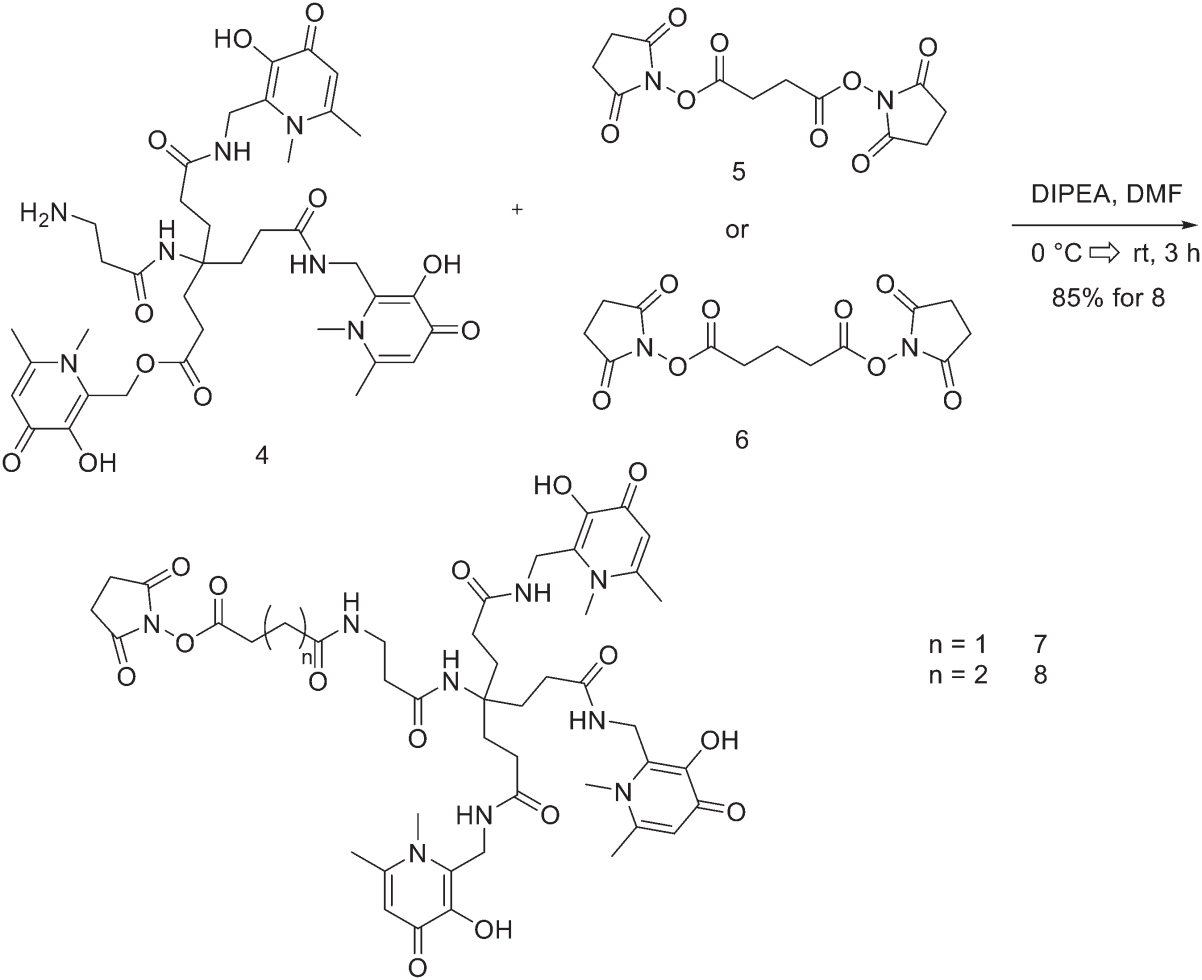
Synthetic scheme for THP-succinic (7) and THP-glutaric (8).

A similar approach was used to obtain H_3_THP-PhNCS: an excess of *p*-phenylene diisothiocyanate and DIPEA in DMF was added to a solution of 1, followed by the formation of the isotiocyanate which was purified by semipreparative RP-HPLC (81% yield). The benzyl groups were removed by using BCl_3_ in DCM, followed by the addition of MeOH, yielding the bifunctional chelator H_3_THP-PhNCS (58% yield). The αvβ3 integrin-targeting pentapeptide, cyclic(RGDfK), was selected as a peptide targeting vector for conjugation to the THP chelators. Both H_3_THP-NCS and H_3_THP-PhNCS were reacted with the primary amino group of the lysine side chain of RGD under microwave-assisted synthesis. Both conjugates H_3_THP-NCS-RGD and H_3_THP-PhNCS-RGD were obtained in 98% purity, after semipreparative HPLC purification. The ^68^Ga-radiolabelling of H_3_THP-NCS-RGD and H_3_THPPhNCS-RGD was performed at RT by the addition of acidic solutions of ^68^Ga^3+^ produced by the generator to solutions of the conjugates, followed by the addition of ammonium acetate. Radiochemical yields were >95% and specific activities of 60–80 MBq nmol^−1^. ^68^Ga^3+^-labelled THP peptide conjugates showed to retain affinity for the αvβ3 integrin receptor and to undergo receptor-mediated tumour uptake *in vivo*. Young *et al.* synthesised a conjugate of a THP chelator with the urea-based PSMA inhibitor and radiolabelled it with ^68^Ga.^90^ At first, the resin bounded PSMA inhibitor was reacted with glutaric anhydride to produce the glutaric acid derivative of PSMA. Then it (1 equiv.) was suspended in a solution of HATU (1 equiv.) and DIPEA (2 equiv.) in dry DMF, and the activation of the carboxylate was allowed to proceed for 10 min after which a solution of THP-NH_2_ (1 equiv.), in DMF/DMSO containing DIPEA (1 equiv.) was added and stirred for 36 h. After cleavage and removal of the sidechain protecting groups, the filtrate was collected, and the resin was washed with TFA and DCM. The solution was concentrated, and ice-cold diethyl ether was added to precipitate THP-PSMA which was purified by semipreparative RP HPLC to give a TFA salt. The yield of THP-PSMA(CF_3_COO)_3_ was 5.2% from resin loading. Radiolabelling yields were >95%, the radiochemical purity was of 95% and specific activity of 15–45 MBq nmol^−1^. *In vivo*, ^68^Ga THP-PSMA accumulated in PSMA-expressing tumours, with good tumour-to-background ratio delineation of PSMA-positive tumour lesions like ^68^Ga-HBED-CC-PSMA. However, it showed easier radiolabelling with respect to other ^68^Ga-PSMA bioconjugates. More recently, Floresta *et al.* developed and synthesised NHS-functionalised THP-derivative.^91^ NHS ester-activated compounds react with primary amines under physiological conditions to slightly alkaline conditions to yield stable amide bonds. The reaction only releases NHS and does not have the limitation of the already proposed THP derivatives, namely THP-maleimide and THP-isothiocyanate (*i.e.*, presence of a thiol group, partial reduction of cysteine in antibodies and/or water instability). The more stable six-carbon-atom product was selected for a proof-of-concept conjugation and radiolabelling study with a synthesised glucagon-like peptide-1 (GLP-1) targeting peptide using solid-phase synthesis. The synthesis of hexadentate tris(3,4-hydroxypyridinone) (1 in Scheme 9) was already reported.^89^ Once the synthesis of 1 was completed, the compound was at first deprotected with an excess of BCl_3_ and then conjugated to the bis-NHS-succinic acid ester (5) and bis-NHS-glutaric acid ester (6) synthesised as previously reported in literature (Scheme 10).^92^ DIPEA (6 equiv.) was added to compound 4 (1 equiv.) in 1 mL of DMF and then this solution was added dropwise into a solution of bis-NHS acid ester derivative (5 or 6) (10 equiv.) in DMF at 0 °C. All the reactions were completed within 3 h. Reactions were dried under vacuum and chromatographed *via* preparative HPLC/UV giving the activated ester THP-glutaric (8) (85% yield), only the hydrolysed product was isolated for molecule 7.

The precursor GLP-1-peptide-THP linear sequence was synthesised using a standard Fmoc solid-phase peptide synthesis approach. After the removal of the Dde group from the lysine, the NHS-THP 8 was site-specifically (40Lys) coupled under mild conditions using 5 equiv. of 8 in DMF by shaking the solution for 12 h without the use of activation agents. After the coupling of the hexadentate tris(3,4-hydroxypyridinone) 8, the peptide was removed from the resin. After precipitation into cold diethyl ether, the crude product was analysed by RP-HPLC and mass spectrometry. The developed NHS-THP chelator could be used for simple, efficient labelling of ^67^Ga/^68^Ga biomolecules under suitable conditions for peptides and proteins in the aqueous phase and with easy synthetic procedures. In a recent study by Floresta and co-workers,^93^ the NHS-THP chemistry was further explored by developing two analogues with two different linkers: bis-NHS-glutaric acid ester and bis-NHS-adipic acid ester. The THP-based compounds were then conjugated to a cyclic peptide that targets the αvβ6 integrin to discover new ^68^Ga-labelled radiopharmaceuticals effective for imaging tumours *via* αvβ6 integrin. Both THP-NHS ester compounds did not exhibit stability issues since the terminal NHS group was not hydrolysed throughout the production or purifying processes. The two molecules were conjugated to the nonapeptide cyclo-(FRGDLAFp(NMe)K)^94^ (p = D-Pro; (NMe)K = *N*-methyl-Lys) under mild conditions, without the assistance of any activation agent. Integrin αvβ6-peptide-THPs were radiolabelled using ∼30 MBq ^68^Ga and 20 μg of chelator, to give a specific activity of ∼3 GBq μmol^−1^. A purification step was not required after metal complexation/radiolabelling. This confirmed the advantage of using THP over other chelators, ensuring to obtain near quantitative radiochemical yields, which can be achieved at low concentrations of chelator, obviating the need to remove unreacted ^68^Ga; the radiochemical yield for both products was >95% (determined by radio-HPLC). Cellular binding experiments demonstrated nanomolar affinity of the probes on BxPC-3 cells, highlighting their potential for *in vivo* monitoring of the transmembrane receptor αvβ6 integrin.

## Design and strategies for ^64^Cu marked peptides

Cu(ii), over the different oxidation states of Cu, bears optimal characteristics for radiopharmaceutical approaches.^52^ The design of copper radiopharmaceuticals is based on polyaza-macrocycles derived BFCAs (Fig. 4). Cyclen and cyclam modified with *N*-acetic acid arms (especially DOTA) are the most common macrocycles used to chelate Cu(ii) for PET imaging. However, the low stability of [^64^Cu]Cu-DOTA-labelled biomolecules under *in vivo* conditions and the high liver accumulation, led to the development of other classes of chelating agents to form more stable ^64^Cu-complexes.

### NOTA derivatives

**Fig. 6:**
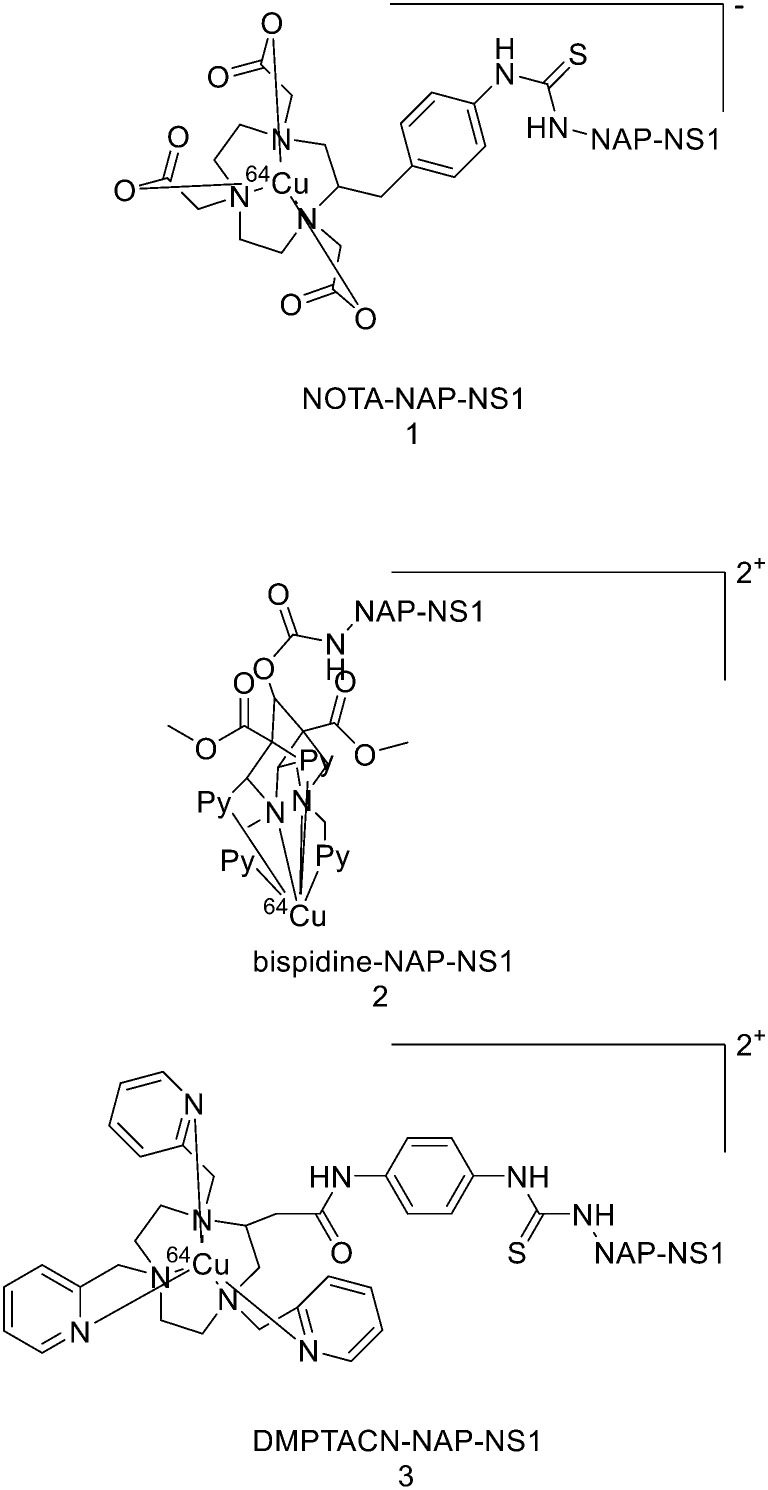
Chemical structures of the ^64^Cu-labelled peptide conjugates.

Recently, NOTA derivatives were studied as chelating agents of ^64^Cu and showed good performance with high labelling yields and good stability *in vivo*. Craft *et al.*^95^ conjugated *p*-SCN-Bn-NOTA to the eight C-terminal amino acids of bombesin [BN(7–14)] in order to form a six-coordinate complex with ^64^Cu against the five-coordinate copper complex of [NO2A-Aoc-BN(7–14)], in which NOTA was conjugated to Aoc-BN(7–14) through one of the carboxylate groups of NOTA.^96^ The *p*-SCN-BN-NOTA (3 equiv.) was coupled to Aoc-BN(7–14) (1 equiv.) in sodium bicarbonate solution (1.4 M) and DMF at RT for 15 h. Then the crude product was purified by preparative HPLC to afford the desired compound (30% yield). The BN analogue was radiolabelled with ^64^Cu at RT for 45 min in >95% radiochemical purity and with a specific activity of 12.4 GBq μmol^−1^. The conjugate had rapid tumour uptake and generally better tumour/normal tissue ratios at early time points after injection. However, at 24 h this improvement was lost, suggesting that either this six-coordinate chelator or its metabolites were cleared from the tumour more easily than the previously described five-coordinate systems. Dumont *et al.* used chelators NODAGA and 4,11-bis(carboxymethyl)-1,4,8,11-tetraazabicyclo[6.6.2]hexadecane (CB-TE2A) to radiolabel the cyclic pentapeptide c(RGDfK) with ^64^Cu.^97^ For the synthesis of NODAGA derivative, after the ivDde group removal, the protected cyclic peptide was reacted with the prochelator 1-(1-carboxy-3-carbo-*tert*-butoxypropyl)-4,7-(carbo-*tert*-butoxymethyl)-1,4,7-triazacyclononane (NODAGA(*t*Bu)_3_) (synthesised according to the literature,^98^ 1 equiv.), together with 1 equiv. of HATU and 2 equiv. of DIPEA in DMF for 3–4 h. All the sidechain protecting groups were then removed with a mixture of TFA/thioanisol/triisopropylsilan/water 95/3/1/1. The resulting product was purified by preparative HPLC. NODAGA-c(RGDfK) was labelled at RT in 10 min, while CB-TE2A-c(RGDfK) was labelled at 95 °C for up to 30 min. The conjugates were labelled with a radiochemical purity >97% and specific activities of 15–20 GBq mmol^−1^, and the radiochemical yields for both radiotracers were ≥97%. Labelled CB-TE2A- or NODAGA-RGD peptides showed an improved image contrast due to the better biodistribution in normal organs. Moreover, NODAGA could be labelled rapidly and under mild conditions. More recently, Gao *et al.* conjugated the α-melanocyte-stimulating hormone (α-MSH)-derived peptide NAP-NS1 with a β-Ala linker (ε-Ahx–β-Ala–Nle–Asp–His–d-Phe–Arg–Trp–Gly–NH_2_) to different chelators: either to *p*-SCN-Bn-NOTA, to a hexadentate bispidine carbonate derivative (dimethyl-9-(((4-nitrophenoxy)carbonyl)oxy)-2,4-di(pyridin-2-yl)-3,7-bis(pyridin-2-ylmethyl)-3,7-diazabicyclo[3.3.1]nonane-1,5-dicarboxylate), or to DMPTACN (*p*-SCN-Ph-bis(2-pyridyl-methyl)-1,4,7-triaza-cyclononane), labelled with ^64^Cu.^99^ The bispidine carbonate derivative and SCN-DMPTACN were synthesised according to literature.^100–102^ For the preparation of the chelator-peptide conjugates (Fig. 6), the peptide NAP-NS1 (1 equiv.), SCN-NOTA (1 equiv.) and TEA (10–15 equiv.) in DMF, were mixed and incubated at 22 °C overnight to obtain NOTA-NAP-NS1 (1). Then, the reaction was quenched by the addition of water. The same procedure was used to afford bispidine-NAP-NS1 (2) and DMPTACN-NAP-NS1 (3). After conjugation, the crude chelator-peptide conjugates were purified by semipreparative HPLC. After purification, the peptide conjugates were obtained with yields of 35%, 29%, and 21%, respectively and purity >98%. The pH and the temperature strongly influenced the labelling with ^64^Cu giving radiochemical yields ranging from 80% and 90% at pH of 6.2 to 6.8. An almost complete complexation at 22 °C was achieved for the three conjugates. However, increasing the temperature to 50 °C reduced the labelling time. The molar activities ranged from 12 to 45 GBq μmol^−1^. The three ^64^Cu-labelled conjugates were stable in buffer and human serum and showed high hydrophilicity and high binding affinity to MC1R in murine and human melanoma cells.

### Cyclam derivatives

**Fig. 7:**
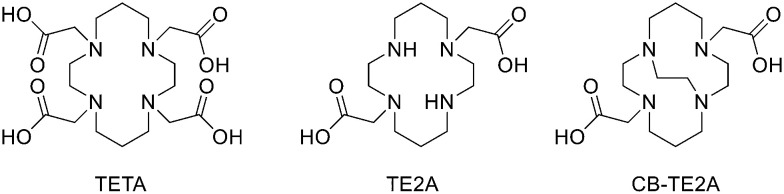
Structures of TETA and its derivatives TE2A and CB-TE2A.

Slightly higher stability against transchelation could be achieved when 1,4,8,11-tetraazacyclotetradecane-1,4,8,11-tetraacetic acid (TETA, Fig. 7) is employed for complexation, as proved by ^64^Cu-TETA-Y3-TATE, developed by Lewis *et al.*^103^ Y3-TATE (Fmoc–d-Phe–Cys(Acm)–Tyr(O*t*Bu)–d-Trp(Boc)–Thr(O*t*Bu)–Cys(Acm)–Thr(O*t*Bu)) was an octreotide (OC) analogue for binding somatostatin receptors, which differed from standard OC for the Tyr that replaced Phe in the 3-position and for the C-terminal threonine that was an acid rather than an alcohol. The linear, protected Y3-TATE was obtained by SPPS. When the synthesis was complete, the cysteines' Acm-protecting groups were removed by using thallium(iii) trifluoroacetate with the simultaneous disulfide formation to obtain the cyclic peptide. The coupling of TETA to the N-terminus took place by reaction with the tri-*tert*-butyl TETA derivative, in presence of HOBt and HBTU. Tri-*tert*-butyl TETA was synthesised by a variation of the published method.^104^^64^Cu-TETA-Y3-TATE was obtained in 98% radiochemical purity and with specific activities ranging from 0.5 to 2.5 mCi mg^−1^ (18–65 MBq μg^−1^) (labelling time: 1 h at 37 °C). This new OC analogue exhibited significantly greater uptake in somatostatin-rich tissues in two tumour-bearing animal models.

Recently, it was demonstrated that TE2A (1,8-*N*,*N*′-bis-(carboxymethyl)-1,4,8,11-tetraazacyclotetradecane) is able to form more stable Cu(ii) complexes than TETA (Fig. 7).

**Scheme 11:**
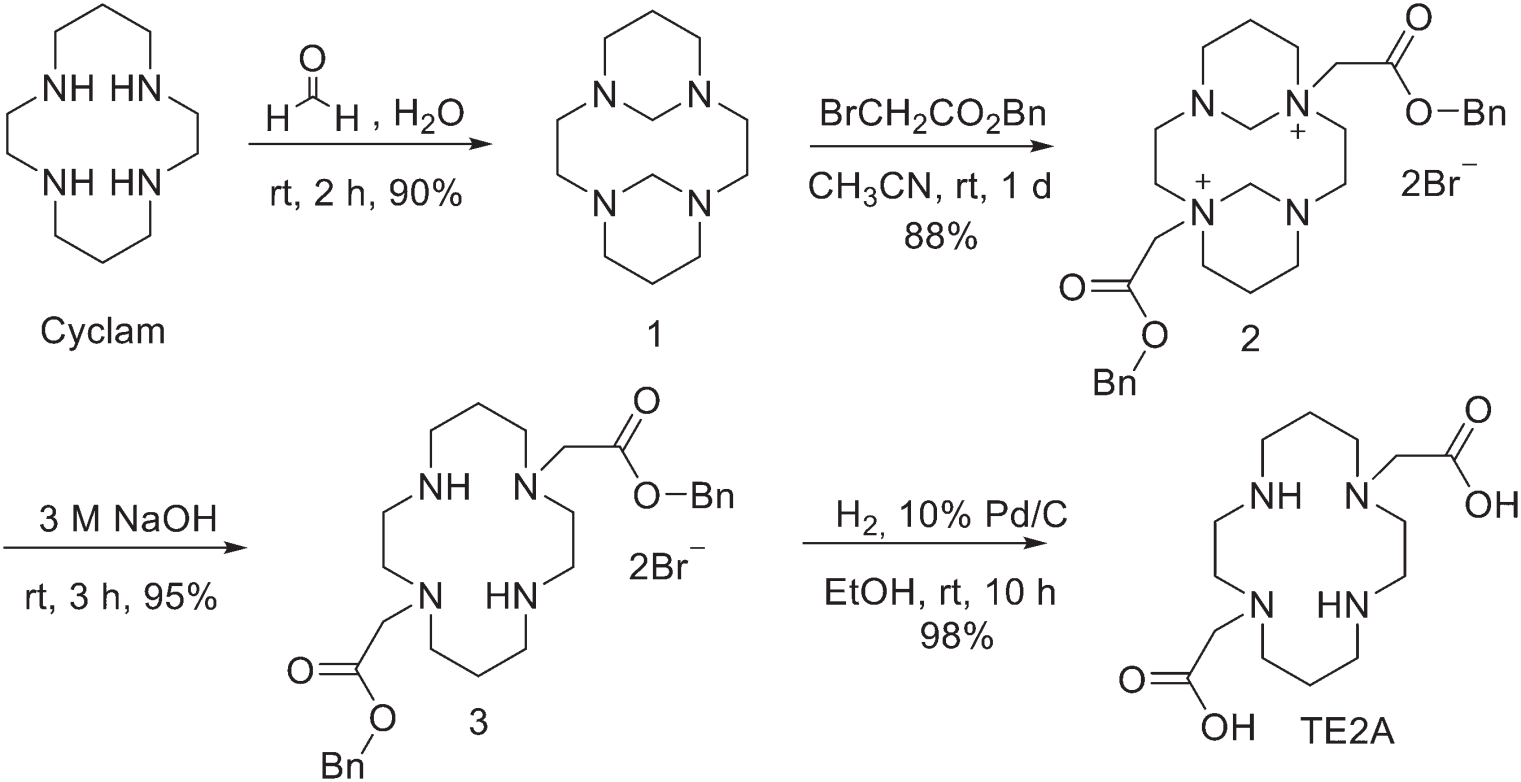
Synthesis of salt-free TE2A from cyclam.

**Scheme 12:**
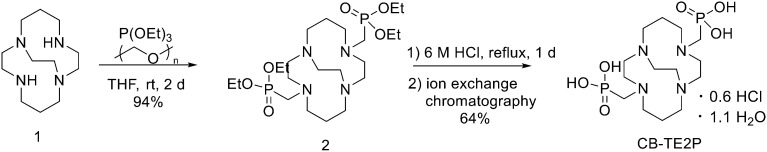
Synthesis of CB-TE2P.

Unfortunately, since it has only two carboxylate groups, the conjugation of TE2A with the amino group of biomolecules is not effortless, in fact, upon conjugation, one of the two carboxylate group would become an amide reducing its binding ability. Furthermore, the two reactive NH of TE2A would compete with the amino group of peptides after activation of the carboxylic acid group of TE2A. Pandya *et al.* reported a new synthetic procedure to obtain salt-free TE2A and demonstrated that TE2A can be coupled to small peptides and radiolabelled with ^64^Cu in high yield.^105^ The synthesis started from commercially available cyclam (Scheme 11); the bisaminal compound 1 was synthesised by the modification of the previously reported method.^106^ Then, benzyl bromoacetate (4 equiv.) was added to a stirred solution of 1 (1 equiv.) in MeCN and the reaction mixture was stirred at RT for 24 h. After filtration of the precipitate, the crude product was crystallized from EtOH to give 2 as a white solid (88% yield). This high yield was due to the optimized conditions where 4 instead of 2 equiv. of benzyl bromoacetate were used and MeCN proved to be the most efficient solvent, while others, such as THF and CHCl_3_ gave modest yields. The methylene bridge of 2 was cleaved by treating it with a 3 M NaOH solution and then the debenzylation was conducted in dry EtOH in presence of 10% Pd/C (98% yield). After that, TE2A (1 equiv.) was activated by EDC (0.5 equiv.) and SNHS (0.6 equiv.) and reacted with cyclic RGDyK peptide (0.06 equiv.) overnight at 4 °C. The TE2A-RGD conjugate was purified by semipreparative HPLC and lyophilised. TE2A-c(RGDyK) conjugate was radiolabelled with ^64^Cu in high yield (87 and 94% in pH 5.5 and 8 buffer solutions), within 30 min at 50 °C; demonstrating that TE2A could be used as a potential bifunctional chelate. The *in vivo* stability of Cu(ii) was increased thanks to the introduction of 1,8-ethylene cross-bridged macrocyclic chelators into ^64^Cu bioconjugates. Among several cyclen and cyclam-based cross-bridged macrocyclic chelators, the most promising one may be 4,11-bis(carboxymethyl)-1,4,8,11-tetraazabicyclo[6.6.2]hexadecane (CB-TE2A, Fig. 7) which displayed higher *in vivo* stability respect to DOTA and TETA. The problem with this chelator is the strong labelling conditions, which allow its use only for heat-insensitive peptides.^107^ More recently Pandya *et al.* synthesised a propylene cross-bridged TE2A (PCB-TE2A) which exhibited high *in vitro* and *in vivo* stability and could be labelled under milder conditions than the corresponding ethylene cross-bridged (ECB-TE2A).^108^ PCB-TE2A was obtained starting from cyclam through transalkylation/cross-bridge reaction/deprotection steps. The regioselective *trans*-disubstitution of the *tert*-butyl acetate groups on the nonadjacent N-atoms of cyclam was achieved in quantitative yield through, at first, the bisaminal protection of four N-atoms and then by the alkylation with 4 equiv. of *tert*-butyl bromoacetate groups in MeCN for 1 day. The bisaminal bonds of this compound were selectively cleaved by using 3 M NaOH to obtain the *trans*-disubstituted cyclam in quantitative yield. Then, a mixture of the disubstituted cyclam and 1,3-propanediol di-*p*-tosylate was refluxed in dry toluene in the presence of K_2_CO_3_. The tosylate counteranion was removed by treatment with 20% NaOH, and the crude product was purified to yield the salt-free form of the compound in 70% yield. The deprotection of the *tert*-butyl ester groups of the intermediate was conducted by using 6 M HCl to give the final product, PCB-TE2A, in the form of hydrochloric salt (99%). PCB-TE2A was radiolabelled with ^64^Cu in 0.1 M sodium acetate buffer adjusted to pH 8, in 89% yield at 70 °C within 10 min, and the labelling yield reached 100% after 1 h. However, the radiolabelling of ECB-TE2A under the same labelling conditions gave low radiochemical yield and purity. One hour of incubation of PCB-TE2A with ^64^Cu at 50 °C yielded ^64^Cu-PCB-TE2A in 89% yield, and the labelling yield reached 58% after 90 min of incubation, even at 40 °C. In contrast, the labelling yield was only 25% at 40 °C after 90 min incubation of ECB-TE2A with ^64^Cu. Biodistribution studies strongly indicated that the ^64^Cu complexes of PCB-TE2A cleared out rapidly from the body with minimum decomplexation. The phosphonate-armed chelators, such as CB-TE2P and CB-TE1A1P, were developed to find a chelator labellable at lower temperatures. Stigers *et al.* described the synthesis of a di-methanephosphonate pendant-armed cross-bridged cyclam, (Scheme 12).^109^ Parent cross-bridged cyclam was, at first, transformed to bis-diethylphosphonate 2 by a modification of the Kabachnik–Fields three-component reaction^110^ and this intermediate was then hydrolysed to obtain the desired ligand. The reaction of 1 with triethylphosphite and paraformaldehyde in dry THF under nitrogen at RT for 4 days gave 2 in 94% yield after workup. Compound 2 was hydrolysed in 6 M HCl under reflux for 24 h to give CB-TE2P with 64% yield as hydrochloride after purification by ion-exchange chromatography. No-carrier-added ^64^Cu-CB-TE2P was prepared in water by the addition of a solution of ^64^CuCl_2_ in 0.1 M HCl to a 2–5 mM solution of the ligand in water. However, a 100% radiochemical yield was observed only at temperatures >90 °C. Under carrier-added conditions, ^64^Cu-CB-TE2P was obtained in a 100% yield even at RT. The biodistribution studies demonstrated that the radiotracer was rapidly excreted through the kidneys. The affinity of the methanephosphonic pendant arms for the hydroxyapatite in the bone was probably responsible for an important percentage of radioactivity at earlier time points. However, this activity was cleared several-fold by 24 h post-injection. In any case, the current use of CB-TE2P as a BFCA using standard bioconjugation procedures is less effortless.

**Fig. 8:**
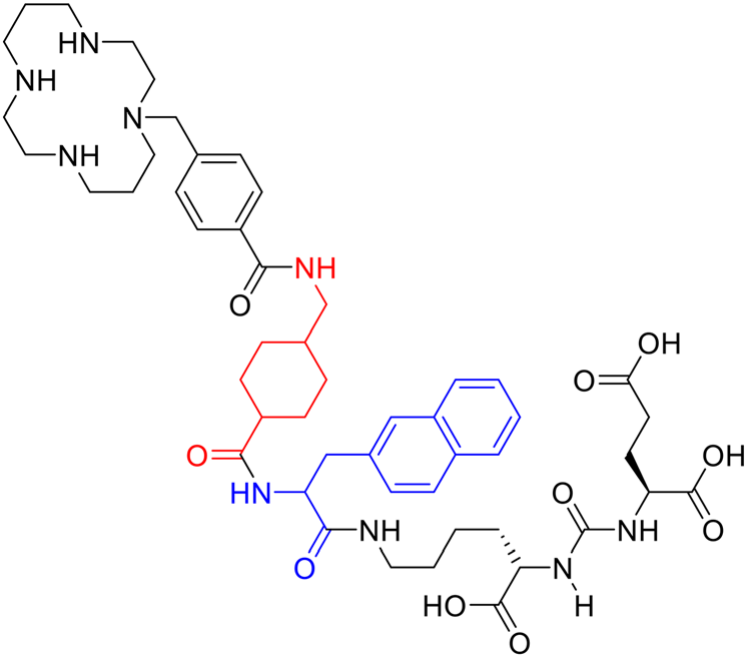
Structure of the most promising ligand suitable for ^64^Cu labelling, bound to PSMA *via* 2-naphthyl-l-alanine and 4 aminomethyl-(cyclohexane)carboxylic acid.

Ferdani *et al.* synthesised a mixed-armed variation, CB-TE1A1P (1,4,8,11-tetraazacyclotetradecane-1-(methanephosphonic acid)-8-(methanecarboxylic acid)), carrying both a methanephosphonic acid as well as a carboxymethyl pendant arm.^111^ CB-TE1A1P was synthesised starting from CB-TE1A *tert*-butyl ester (1 equiv.) which reacted with triethylphosphite (1.8 equiv.) and paraformaldehyde (1.8 equiv.) in dry THF for 4 days at RT to furnish the corresponding phosphonate ester, which was used for the next step without further purification. Hydrolysis of both the diethylphosphonate and *tert*-butyl carboxylic ester moieties in refluxing 6 M HCl furnished the final compound in 62% overall (two-step) yield as a hydrochloride salt after purification by ion exchange chromatography. Radiolabelling of CB-TE2P and CB-TE1A1P with ^64^Cu were conducted under aqueous conditions with various buffers, pH values (4.5–8.1), and temperatures (25–95 °C). The optimized conditions resulted in the incubation of 100 μL of a 26.5 μM solution of chelator in 0.1 M NH_4_OAc (pH = 8.1) with 1 mCi of ^64^CuCl_2_ at RT. Radiochemical yields >95% were achieved after 30 min (specific activities >1 mCi μg^−1^). ^64^Cu labelling of CB-TE2A under the same conditions resulted in negligible radiochemical yields. Biodistribution studies using healthy male Lewis rats served to determine the *in vivo* behaviour of both ^64^Cu-CB-TE2P and ^64^Cu-CB-TE1A1P. Their biodistribution was similar, apart from a lower accumulation in the bone at all time points (*p* < 0.0001). Both compounds displayed rapid clearance with comparable or lower accumulation in non-target organs/tissues with respect to other copper chelators including CB-TE2A and NOTA. However, the *in vivo* stability with ^64^Cu-labeled chelator-bioconjugates could not be predicted by these results with ^64^Cu chelates, in fact once that CB-TE1A1P is linked to a biomolecule, the carboxylate group would be converted into an amide group. It has already been demonstrated that the amide oxygen reduced *in vivo* stability with respect to the carboxylate moiety, and this reduced the stability of a CB-TE1A1P-bioconjugate. dos Santos *et al.* developed many different tracers made out of copper chelators based on the TETA scaffold, combined with the PSMA-specific binding motif.^112^ These new chelators were obtained in high yields and characterized by LC–MS and were labelled with ^64^Cu with an excellent radiolabelling yield (>98%). The tracers differed for the presence of acid functionalities linked to one or more N atoms or by the cross bridge. These chelators were conjugated to the PSMA-binding motif either *via* 2-naphthyl-l-alanine or *via* 2-naphthyl-l-alanine and the additional spacer 4-aminomethyl-(cyclohexane)carboxylic acid. The products were obtained by incubating the resin with 1.5 equiv. of chelator NHS ester derivatives and 10 equiv. of DIPEA in DMF. Subsequently, the PSMA coupled to the chelator was cleaved from the resin. For the radiolabelling with ^64^Cu, the conjugates were added to a mixture of 400 μL of sodium acetate buffer (0.4 M in water, pH 5.0), 10 μL of ascorbic acid (20% in water), and 282 μL of ^64^CuCl_2_ in 0.1 M HCl (200 MBq). The mixture was heated at 95 °C for 5 min. Radiolabelling of 0.2 nmol of the precursor at 95 °C with ^64^Cu led to yields >98% within 10 min. The specific activity of the most promising compound was approximately 40 MBq nmol^−1^. Of the ^64^Cu-labelled PSMA-ligands obtained, the compound shown in Fig. 8 demonstrated high potential affinity, high PSMA-specific uptake, fast clearance, and rapid kidney excretion.

### Sarcophagine-based chelators

**Fig. 9:**
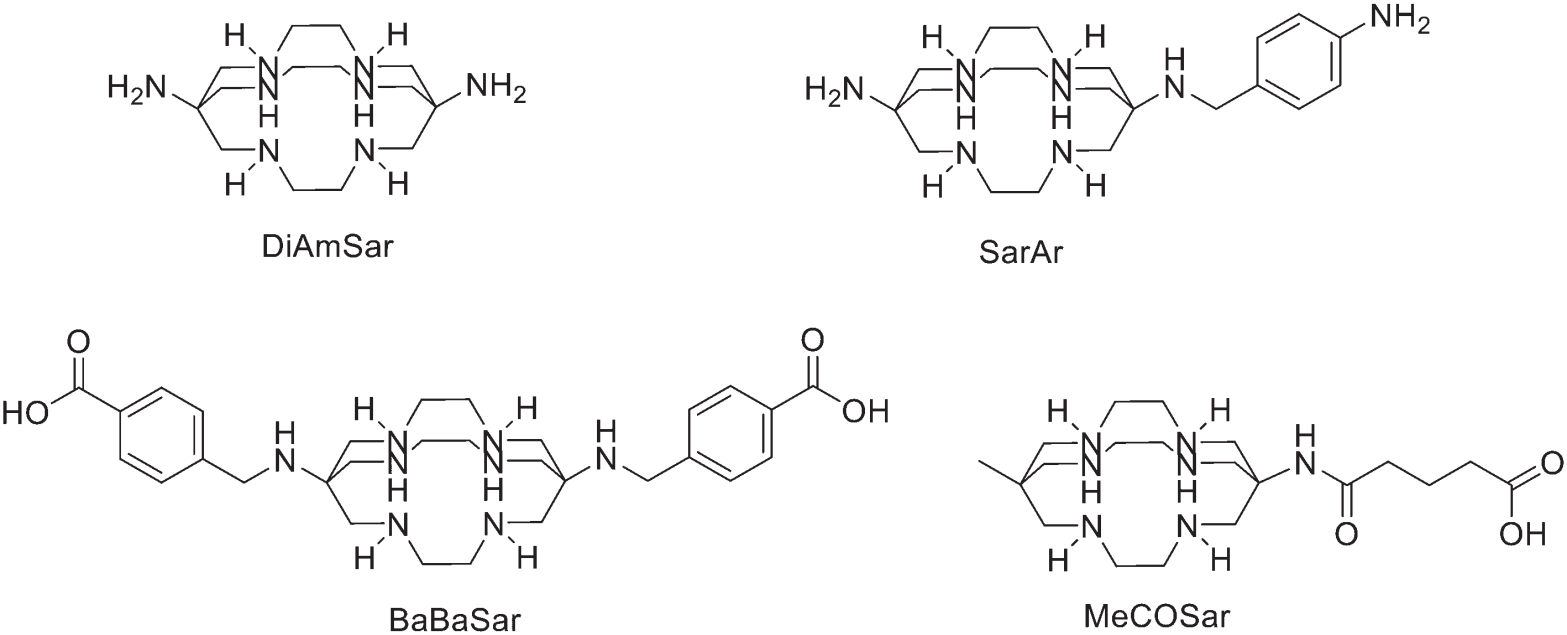
Chemical structures of sarcophagine (Sar)-based chelators.

Hexaazamacrobicyclic cage-type chelators, namely ligands based on sarcophagine scaffold, are used as BFCAs for labelling with ^64^Cu. These chelators allow fast labelling at RT with the formation of very stable complexes. The simplest structure is the one of DiamSar (diaminosarcophagine, 1,8-diamino-3,6,10,13,16,19-hexaazabicyclo[6.6.6]icosane); but the direct coupling of the primary amino groups of the cage with peptides using conventional coupling methods is not straightforward. Another one is SarAr which is the Diamsar chelator bearing a linker; it shows higher labelling efficiency after coupling.^113^ The lipophilicity of these compounds led to the introduction of cationic or natural Cu-complexes. For this reason, derivatives like BaBaSar, containing carboxylate moieties, have been developed (Fig. 9).

Liu *et al.* conjugated BaBaSar chelator to RGD peptide.^114^ They started from hexaazamacrobicyclic sarcophagine which was directly alkylated with 4-bromomethylbenzoic acid to obtain BaBaSar in 36% yield. The monoalkylated derivative (AmBaSar) was also isolated in a 30% yield. After they synthesised the bi-functionalised BaBaSar, EDC/SNHS were employed to activate its free carboxylic acids, and then it was coupled to c(RGDyK) in the presence of DIPEA. After HPLC purification, BaBaSar-RGD_2_ was afforded in 78% yield. The BaBa-Sar-RGD_2_ was labelled with ^64^Cu in 0.1 M NH_4_OAc buffer within 5 min at RT. The radiochemical yield was 90.7 ± 5.1% (*n* = 4). The specific activity of ^64^Cu-BaBaSar-RGD_2_ was estimated to be 200–500 μCi μmol^−1^ (5.4–13.5 GBq μmol^−1^). ^64^Cu-BaBaSar-RGD_2_ showed high stability both *in vitro* and *in vivo*. The higher tumour uptake of ^64^Cu-BaBaSar-RGD_2_ compared to its ^64^Cu-AmBaSar-RGD_2_ analogue highlighted the advantages of the BaBaSar structure. More recently, they evaluated the biodistribution of ^64^Cu-BaBaSar-RGD_2_ in monkeys with PET and estimated the absorbed doses in main organs for humans. The study demonstrated the promising use of ^64^Cu-BaBaSar-RGD_2_ as an integrin marker, with good biodistribution and safety properties in monkeys.^115^ Ma *et al.* derivatised both of the primary amines of DiamSar with succinic or glutaric anhydride and linked the chelators with Lys^3^-bombesin.^34^ The functionalisation of DiamSar could be difficult due to the presence of eight amino groups. An interesting procedure to “protect” the six secondary amino groups is the metal ion coordination, which allows selective couplings with the terminal primary amino groups.^116^ Here Cu(ii) coordination was employed to protect the secondary amines of DiamSar from reaction with both the anhydrides, giving a selective acylation on the primary amines. For the synthesis of the acylated diamino sarcophagine compounds, a solution of [Cu(NH_3_)_2_sar](CF_3_SO_3_)_4_ (1 equiv.) in dry DMA was heated under a nitrogen atmosphere at 70 °C. Succinic anhydride or glutaric anhydride (1.2 equiv.) and DIPEA were added and the solution was heated at 70 °C for 2 h. After cooling, the solution was purified to give the desired compounds. Even if stoichiometric equivalents of anhydride were used, both the monoacylated and the diacylated derivatives were observed. The major formation of the diacylated compound was achieved using an excess of cyclic anhydride (>2 equiv.). After that, the reaction between an excess of sodium sulfide and the Cu(ii) used to protect the secondary amines, allowed its removal from the ligand. The four secondary amines of the obtained ligands were protected by reaction with ditert-butyldicarbonate and DIPEA in MeCN. The *N*-hydroxysuccinimide esters of (*t*-Boc) ligands were obtained by the reaction of NHS with (*t*-Boc) derivatives in the presence of DIC. After purification by semipreparative HPLC, Lys^3^-bombesin was obtained using standard SPPS and reacted with the activated esters, to afford a homodimeric sarcophagine conjugate bearing two bombesin moieties. After deprotection and purification by semipreparative HPLC, the peptide conjugates were obtained. The radiolabelling was performed by addition of ^64^CuICl_2_ (19 MBq, 40 μL 0.4 M ammonium acetate, pH 6) to the conjugates (0.2 mg mL^−1^ peptide, 40 μL 0.1 M ammonium acetate, pH 6) at RT (RCY > 99%). The radiolabelling showed to be pH dependent, but, among all the used conditions, the ideal radiolabelling took place at RT and pH 5–7. The new dimeric conjugates gave very stable complexes with ^64^Cu and could overcome the disadvantages of other chelators such as the metal ion loss from the ligand *in vivo*.

More recently, Kelly *et al.* developed a ligand that binds PSMA and serum albumin, the RPS-085 which exploited the ^64/67^Cu radionuclide pair for prostate cancer theranostics. RPS-085 was obtained by a reaction between a PSMA-targeting moiety, a *N*ε-(2-(4-iodophenyl)acetyl)lysine albumin binding group, and the activated ester of MeCOSar chelator.^117^ A mixture of the PSMA ligand (1 equiv.) and DIPEA (10 equiv.), was stirred for 5 min at RT in dry DMF. Then, a solution of the *N*-succinimidyl ester of MeCOSar^118^ (1.2 equiv.) in DMF was added dropwise to the reaction, and the resulting mixture was stirred for 5 h at RT. After purification by HPLC, a white solid was obtained (53% yield). [^64^Cu]Cu-RPS-085 was quantitatively radiolabelled (*n* = 6) in 20 min at 25 °C in both 10 × PBS (pH 7.4) and 0.5 M NH_4_OAc (pH 5.5). At the same temperature, labelling in 3 M NaOAc (pH 4.5) was accomplished in 35 ± 2% yield (*n* = 2). When the starting activity was 800 MBq, [^64^Cu]Cu-RPS-085 was isolated with a molar activity of 117 GBq μmol^−1^ and radiochemical purity >99%. [^67^Cu]Cu-RPS-085 was also quantitatively radiolabelled at a concentration of 6.2 μM in 0.1 M NH_4_OAc in 20 min at 25 °C. The pH of the reaction mixture was approximately 6. With a starting activity of 245 MBq, [^67^Cu]Cu-RPS-085 was isolated with a molar activity of 41 GBq μmol^−1^ and a radiochemical purity >99%. The study showed that RPS-085 was able to bind PSMA with high affinity and complexed [^64/67Cu^]Cu^2+^ with high *in vivo* stability thanks to its MeCOSar ligand. Moreover, [^64/67^Cu]Cu-RPS-085 demonstrated to give high tumour uptake and excellent tumour-to-background ratios even at early time points.

## Design and strategies for ^89^Zr marked peptides

Zirconium, a metal belonging to group IV of the periodic table, acts as the hard Lewis acid and forms complexes with high coordination number with anionic oxygen donor groups. Due to its long half-life, ^89^Zr has been employed especially in the labelling of monoclonal antibodies for PET imaging,^119^ but, more recently, ^89^Zr-labelled small peptide PSMA-inhibitors for prostate cancer imaging have also been developed.^120^

### DFO and its derivatives

**Fig. 10:**
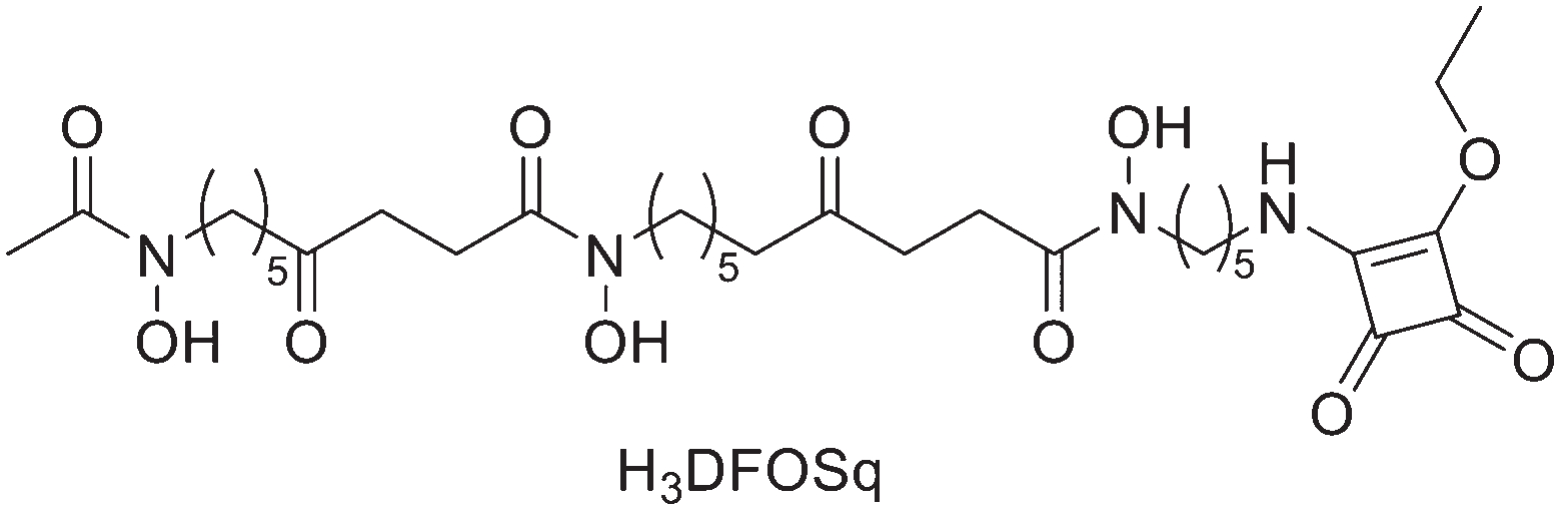
Chemical structure of squaramide ester derivative of desferrioxamine B.

The most common chelating systems for ^89^Zr are based on desferrioxamine B (Fig. 4) and its derivatives. Very recently, Noor *et al.* developed a squaramide ester derivative of desferrioxamine B (H_3_DFOSq, Fig. 10), which is able to form stable complexes with ^89^Zr.^120^

They used H_3_DFOSq to synthesise four new agents with either one or two lysine–ureido–glutamate pharmacophores. Specifically, they prepared two monovalent ligands: H_3_**L**^**1**^, with a hydrophobic aromatic *p*-aminomethylbenzamide linker and H_3_**L**^**2**^ with an aliphatic anionic carboxylate linker group derived from EDTA anhydride. The introduction of the tripodal tetraamine, tris(2-aminoethylamine), furnished two divergent primary amine functional groups allowing the attachment of two Lys–ureido–Glu targeting motifs to give H_3_**L**^**3**^. A second bivalent agent, H_3_**L**^**4**^, was also prepared where each Lys–ureido–Glu motif was separated from the ligand by the same hydrophobic aromatic *p*-aminomethylbenzamide linker used in H_3_**L**^**1**^. The radiolabelling with ^89^Zr was conducted at RT in a time ranging from 5 to 70 min depending on the complex. The radiochemical purities were >95% and radiochemical yields were ≥70%. All four [^89^Zr]Zr(iv) tracers were studied in a PSMA-positive LNCaP xenograft model in mice. The PET images demonstrated that all the compounds have important tumour uptake at 1 h post-injection, but the bivalent tracers, [^89^Zr][Zr**L**^**3**^] and [^89^Zr][Zr**L**^**4**^] displayed higher tumour uptake than the monovalent agents. Very recently, an example of ^89^Zr radiolabelled heterodimeric peptide targeting VEGFR (vascular endothelial growth factor receptor) and integrin was also reported. Liu *et al.*^121^ combined iRGD (CRGDK(Fmoc)GPDC, with high affinity to *α*_v_ integrins) and ^D^A7R (atwlppr, targeting VEGFR_2_ and NRP-1) together, to obtain a specific carrier suitable for ^89^Zr-based immunoPET tumour imaging. They developed a new ^89^Zr labelled radiopharmaceutical (Fmoc-iRGD-PEG_3_-(lys-[^89^Zr]Zr-DFO)-PEG_3_-^D^A7R) and systematically investigated its cell binding affinity and tumour uptake in glioma-bearing mice. For the preparation of DFO-heterodimeric precursors or monomeric peptide precursors, iRGD-PEG_3_-lys-PEG_3_-^D^A7R, iRGD and ^D^A7R (2 μmol) in 20 mL of DMSO with 1% TEA was mixed with 2–3 folds molar excess DFO-Bz-NCS (4–6 μmol), and the mixture was stirred for 2 h at RT. Compounds were purified by RP-HPLC. Radiolabelling of DFO-heterodimeric peptide using ^89^Zr was optimised for three main variables: (1) reaction media (carbonate or Na_2_CO_3_ + 2-[4-(2-hydroxyethyl)-1-piperazinyl] ethane sulfonic acid-HEPES buffer with different pH values), (2) labelling time (30, 60, and 120 min), and (3) precursor amount. Radiolabelling of iRGD and ^D^A7R using ^89^Zr was prepared according to the best labelling method: the solution pH was first adjusted with sodium carbonate and then kept in the range of pH 7–8 by the addition of a small amount of HEPES. As a result, high radiolabelling yield (88.7 ± 2.4%) was obtained. The radiochemical purity was over 95% after purification on the G-10 gel column. In addition, the labelling rate of either monomeric peptide could reach ∼90%, of which the radiochemical purity would be up to ∼95% after purification. *In vitro* experiments showed that the developed tracer had high binding affinity towards selected cancer cells. The microPET/CT imaging of U87MG murine xenograft models clarified the rapid and specific targeting of [^89^Zr]Zr-DFO-heterodimeric peptide in tumour neovascular system. Both the imaging and biodistribution investigation has demonstrated that this new PET probe could be rapidly cleared from the organism through renal excretion.

However, the hexadentate DFO is not ideal for satisfying the Zr^4+^ cation coordination sphere since it is not able to fulfil Zr^4+^ octavalent demands.

Since a macrocyclic system increases complex stability, some efforts have been made to obtain these structures for the complexation of ^89^Zr. These methods include the use of natural siderophores like fusarinine C and their derivatives.

### Fusarinine C (FSC)

Summer *et al.* used fusarinine C (Fig. 4) as ^89^Zr chelating agent for mono-, di-, and trimeric minigastrin (MG) bioconjugates for targeting CCK2R expression.^122^ Acetylation of [Fe]FSC (extracted from fungal culture) was conducted in dry MeOH, in presence of acetic anhydride for 5 min at RT under vigorously stirring. The resulting mixture of mono- and multiple acetylated derivatives was purified *via* preparative RP-HPLC. The functionalisation with maleimide linker was made by reacting [Fe]FSC, monoAcetylated[Fe]FSC and diAcetylated[Fe]FSC in dry DMF in presence of 3-(maleimido)propionic acid (mal) *N*-hydroxysuccinimide ester (2–4 equiv.) and DIPEA (1–3 equiv.) The mixture was stirred for 10 min at RT. The solvent was evaporated and aqueous Na_2_EDTA (100 mM) was added to each vial and stirring was continued overnight. After confirmation of the complete removal of iron by analytical RP-HPLC analysis, the products were isolated by preparative RP-HPLC. Finally, the conjugation of up to three targeting vectors was performed by reacting site-specifically *via* FSC-mal_3_, mAcFSC-mal_2_, and dAcFSC-mal, dissolved in phosphate-buffered saline (PBS, pH 7.2) with the thiolated MG analogue (MG11-SH, 1.5–4 equiv.). After 2 h of continuous stirring at RT, the reaction was complete, and the products were lyophilised and isolated in high purity with yields from 63% to 84%. Mono- and multimeric conjugates were quantitatively labelled with ^89^Zr after 30–60 min at RT at high molar activities and high radiochemical purity. The resulting radiotracers demonstrated highly specific receptor targeting properties which were only partly improved in terms of binding affinity and *in vivo* targeting by the grade of multimerization.

### Other macrocyclic systems

Other attempts to effectively use ^89^Zr were made by using 3-hydroxypyridin-2-one (2,3-HOPO) derivatives^123^ or DOTA derivatives;^124^ but these chelating agents have been applied mostly in the labelling of monoclonal antibodies^125^ than peptides, for which there are very few examples.

## Design and strategies for ^124^I marked peptides

**Fig. 11:**
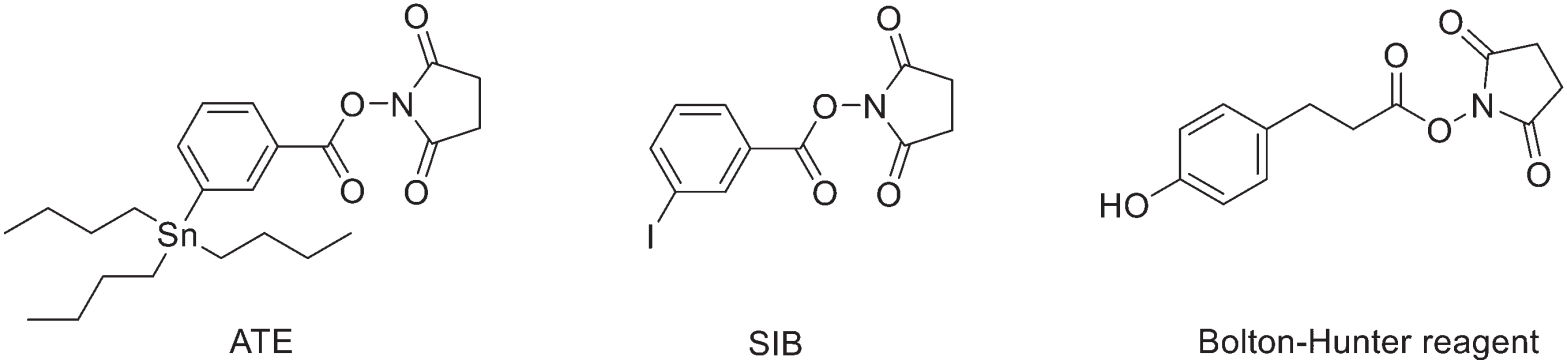
Chemical structure of prosthetic groups ATE, SIB and Bolton–Hunter reagent.

There are different iodine radioisotopes available; of these, ^124^I is the one employed for PET. Radioiodination strategies are the same for all isotopes. The electrophilic substitution of the activated protons in positions 2 and 5 of the phenol ring of tyrosines in the peptide chain, represents the simplest route to label peptides. The process exploits the action of an oxidizing agent to oxidise the iodide to the iodine cation (I^+^). For oxidation, different methods are available, such as the chemical oxidation of iodide with chloramine-T. For example, Vaidyanathan *et al.* synthesised radioiodinated octreotate analogues either through direct radioiodination of peptides, achieved by substitution on the tyrosine residues, or *via* a prosthetic group on which a radioiodine can be installed.^126^ However, direct radioiodination can be susceptible to *in vivo* deiodination. Deiodination can be reduced if a non-phenolic prosthetic group is employed to indirectly radiolabel the biomolecule. For this reason, they used both (*N*-succinimidyl 3-(tri-*n*-butylstannyl)benzoate) (ATE) and *N*-succinimidyl-3-iodobenzoate (SIB) as prosthetic groups and a sugar functional group (Fig. 11). The tri-*n*-butylstannyl group in ATE could be easily replaced by radioiodine. After the cleavage of the peptide from the resin and the removal of the Fmoc protecting group on the Lys^0^, the prosthetic groups ATE or SIB, were conjugated to the amino group of the Lys in presence of TEA in DMF. The reaction mixture was stirred at RT under an inert atmosphere for 18 h. Then, a solution of peptide in EtOH was transferred to a vial and the EtOH was evaporated. To this were added ^131^I and *N*-chlorosuccinimide (in acetic acid) and the mixture was sonicated at RT for 2 min and injected into an analytical RP-column. The labelled peptide was isolated in 21.2 ± 4.9% (*n* = 10) radiochemical yields, radiochemical purity was >90%. These analogues were found to be more resistant to deiodination than (Tyr^3^)-octreotide analogue. For the standard radioiodination, to a solution of sodium [^125^I]iodide, 0.05 M phosphate buffer (pH 7.5) and a solution of peptide in 0.05 M acetic acid were added The mixture was vortexed and chloramine-T in phosphate buffer was added. The labelled peptide was isolated by RP-HPLC, after a 1 min-reaction at RT. If the peptide sequence presents amino acids susceptible to oxidation like Cys, Met or His, which can also be iodinated at the ring system, it is preferred the enzymatic oxidation of iodide with lactoperoxidase. Another strategy, as mentioned above, could be the use of prosthetic group labelling. The most used prosthetic group for iodination is the Bolton Hunter reagent [*N*-succinimidyl-3-(4-hydroxyphenyl) propionate] (Fig. 10).^127^ It is commercially available in the iodinated form, but it could be also easily iodinated using the same strategies reported for the labelling of tyrosine. Thanks to the activated ester, the iodinated Bolton–Hunter can be linked to any amino function in the peptide sequence-*N*-terminally.

## Design and strategies for ^44^Sc marked peptides

Recently ^44^Sc received attention as a potential radionuclide for PET due to its half-life of 3.97 h, which allows the synthesis of many radiotracers with longer pharmacokinetic profiles. Furthermore, the existence of the β-emitting ^47^Sc creates the opportunity to develop new theranostic strategies.^128^

### DOTA derivatives

The most common chelating and labelling strategies for ^44^Sc are based on the use of DOTA. Hernandez *et al.* developed a ^44^Sc-labelled RGD-based peptide for *in vivo* PET imaging of integrin αvβ3 expression in a preclinical cancer model.^129^ The dimeric cyclic-RGD peptide, (cRGD)_2_ (1 equiv.), was directly conjugated to 2-(*p*-isothiocyanatobenzyl)-1,4,7,10-tetraazacyclododecane-*N*,*N*,*N*,*N*-tetraacetic acid (*p*-SCN-Bn-DOTA, 2 equiv.) dissolved in dry DMSO, under basic conditions (PBS, pH 9.0). The reaction was stirred for 2 h at RT. DOTA-(cRGD)_2_ was purified by semipreparative RP-HPLC and lyophilised to afford a white powder. DOTA-(cRGD)_2_ was radiolabelled with ^44^Sc in high yield (>90%) and specific activity (7.4 MBq nmol^−1^). The reaction was incubated at 90 °C for 15 min under continuous stirring. The *in vitro* and *in vivo* binding affinity and specificity studies of ^44^Sc-DOTA-(cRGD)2 for integrin αvβ3 showed to be comparable to previous reports on the use of ^64^Cu-DOTA-(cRGD)2 and ^68^Ga-NOTA-(cRGD)2 in the U87MG tumour model in terms of tumour targeting efficacy, pharmacokinetic profile and PET image quality.^130,131^ As mentioned before, Ferguson *et al.* developed a metabolically stabilised bombesin derivative DOTA-Ava-BBN2 labelled with both ^44^Sc and ^68^Ga as radiopeptide antagonists for PET imaging of GRPRs in breast and prostate cancer.^64^ Also in this case, Ava-BBN2 was derivatised with bifunctional chelator *p*-SCN-Bn-DOTA through the formation of thiourea in solution. Ava-BBN2 (1 equiv.) in DMF was reacted with *p*-SCN-Bn-DOTA (1.3 equiv.) and TEA. The reaction mixture was allowed to proceed at 37 °C for 16 h. Then, peptide DOTA-Ava-BBN2 was purified by HPLC and lyophilised to obtain the desired compound in 85% yield and high chemical purity. Peptide DOTA-Ava-BBN2 was labelled with ^68^Ga and ^44g^Sc at 95 °C for 20 min and then the radiolabelled peptides were purified *via* SPE, eluted from the cartridge with EtOH and isolated by evaporation of the solvent.^68^Ga- and ^44g^Sc-DOTA-Ava-BBN2 were obtained in 70–80% DC radiochemical yields at high radiochemical purity (>97%). Effective molar activities were in the range of 3–8 GBq μmol^−1^ using starting activities of 100–400 MBq for ^68^Ga and ^44g^Sc. [^68^Ga]Ga-DOTA-Ava-BBN2 and [^44g^Sc]Sc-DOTA-Ava-BBN2 showed similar tumour uptake and retention with rapid blood and renal clearance profiles. Both radiopeptides displayed fast clearance of radioactivity from the blood and non-target tissues giving favourable image contrast and high tumour-to-muscle ratios. More recently, Fedotova *et al.* reported the synthesis of an ultra-short somatostatin analogue Thz–Phe–d-Trp–Lys–Thr-DOTA (DOTA-P4) labelled with trivalent cations including ^44^Sc.^132^ The precursor of DOTA (DO3A, 1.1 equiv.) was linked to the chloroacetyl derivative of the peptide (1 equiv.), in presence of dry potash (1.3 equiv.) in DMF and stirred at RT for 3 days. Then the mixture was poured into ice water, the precipitate was filtered off, washed with water and air-dried to obtain the product (95% yield). After the removal of *t*-butyl and Boc groups in presence of TFA and TIBS, the final compound was purified and obtained as a white solid in 6% yield. The labelling of DOTA-P4 with ^44^Sc was conducted in different conditions of pH, incubation time and concentration of DOTA-P4. The optimized conditions (pH 5.2, 0.2 mM of DOTA-P4) resulted in a labelling yield of 94% within 20 min and radiochemical purity greater than 95%. Domnanich *et al.* investigated the use of NODAGA for the complexation of ^44^Sc. They evaluated the *in vitro* and *in vivo* properties of two pairs of DOTA/NODAGA-derivatised peptides and compared the results obtained with ^44^Sc, with the ones obtained for ^68^Ga-labelled analogues. Both DOTA-functionalised peptides were readily labelled with ^44^Sc and ^68^Ga, respectively, and remained stable over at least 4 half-lives of the corresponding radionuclide. In contrast, the ^44^Sc labelling of peptides with NODAGA chelator was more challenging and the resulting radiopeptides were less stable than the DOTA-derivatised counterparts.^133^

### AAZTA derivatives

Very recently, Ghiani *et al.* synthesised a new AAZTA chelator conjugated to PSMA inhibitor labelled with ^44^Sc at mild conditions.^134^ AAZTA–(CH_2_)_4_–COOH tetra *t*-butyl ester^135^ (AAZTA^5^, Fig. 4, 1.5 equiv.) was added to the reaction together with DIPEA (3 equiv.) and HBTU (1.2 equiv.). After 2 h, the resin was washed, and the protected peptide was cleaved from the resin with a solution of HFIP/DCM 1/4 for 5 min. Next, the PSMA-binding motif conjugation was carried out in solution. The final purification performed by semi-preparative HPLC gave the final compound with a total yield of 9% and a purity of 97%. This new derivative for PSMA showed to be a promising ligand for the labelling with ^44^Sc at RT with short reaction time (5 min, pH = 4, RCY >95%) using 1 μM solution of ligand. Furthermore, at high temperatures, a smaller amount of ligand respect to DOTA could be employed to obtain labelling solutions with higher molar activity.

Sinnes *et al.* used the same bifunctional AAZTA^5^ derivative coupled to the PSMA-617 backbone on solid phase and radiolabelled with ^44^Sc (and with ^68^Ga and ^177^Lu, too).^79^ The standard amide coupling using HBTU/HOBt and DIPEA was employed to conjugate the AAZTA^5^ to PSMA. AAZTA^5^(*t*Bu)_4_ (1 equiv.) was reacted with HATU (1 equiv.), HOBt (2.8 equiv.) and DIPEA (2.8 equiv.) in dry DMF (2 mL) and stirred for 20 min. The solution was added to PSMA-617 (resin) (0.7 equiv.) soaked in dry DMF and shaken overnight at RT. Then, after the cleavage from the resin with TFA, the solution was concentrated under vacuum and purified *via* HPLC to afford a white solid (12% yield). AAZTA^5^-PSMA-617 was radiolabelled in quantitative radiochemical yields (>99%) in less than 5 min at RT. [^44^Sc]Sc-AAZTA^5^-PSMA-617, in combination with the faster and milder radiolabelling features, has proven to be a promising radiopharmaceutical for *in vivo* application in prostate cancer.

## Design and strategies for ^76,77^Br marked peptides

Bromine-76 and bromine-77 are positron emitters with a half-life of 16.2 h and 57.0 h, respectively. The introduction of a radiobromine in a biomolecule could be achieved with the same methods of iodine using the electrophilic substitution approach on tyrosine in the peptide sequence facilitated by an oxidation agent, such as chloramine-T, or facilitated by enzymes. Moreover, prosthetic group labelling methods can be employed, as for example, Bolton Hunter reagent.^136^

A common approach is the installation of a radiobromine molecule at any position of the benzene ring using a bromodestannylation reaction from corresponding precursor with a trialkylstannyl group, such as [^77^Br]*N*-succinimidyl 3-bromobenzoate ([^77^Br]SBrB) from ATE (Fig. 11). For example, Ogawa *et al.* prepared radiobrominated (and radioiodinated) peptides ^77^Br-c(RGDyK) and [^77^Br]SBrB-c(RGDfK) or [^77^Br]SBrB-EG2-c(RGDfK), which had an ethylene glycol linker, by the introduction of radioisotope into the tyrosine residue of c(RGDyK) employing the chloramine-T method or by indirect labelling using standard electrophilic halogenation of the tri-*n*-butylstannyl group of ATE.^137^ For the synthesis of [^77^Br]SBrB-c(RGDfK) and [^77^Br]SBrB-EG2-c(RGDfK), at first [^77^Br] SBrB was obtained by reaction of ATE with chloramine-T (1 mg mL^−1^) in EtOH, 1% acetic acid in EtOH, and ^77^Br^−^ (3.7 MBq) at RT for 10 min. After purification of [^77^Br]SBrB by RP-HPLC, it was reacted respectively with c(RGDfK) or NH_2_-EG2-c(RGDfK) in presence of TEA in DMF for 40 min at RT. However, the radiochemical yields and the biodistributions of RGD peptides labelled with ATE were not suitable for tumour imaging and radionuclide therapy, even if ethylene glycol was introduced, compared with that of RGD peptides with direct labelling on the tyrosine residue.

Lang *et al.* developed a new labelling compound, *N*-succinimidyl-3-[^76^Br]bromo-2,6-dimethoxybenzoate ([^76^Br]SBDMB) for cyclic RGD peptide labelling.^13^ The electron-rich 2,6-dimethoxybenzene ring (DMB) could easily facilitate the insertion of ^76^Br at its activated position *ortho* to the methoxy group without the presence of a leaving group. For this reason, *N*-succinimidyl-2, 6-dimethoxybenzoate was also used to pre-attach the DMB moiety to the peptide, which could then be labelled with ^76^Br. Thus, it could be used either for one-step labelling through pre-conjugation or for indirect labelling as a labelled prosthetic group. The conjugation between the amino group of the lysine of c(RGDfK) and [^76^Br]SBDMB took place in 1 M borate buffer (pH 8.4) in presence of ^76^Br prosthetic group at RT for 10 min. Then the mixture was purified by RP HPLC. ^76^Br labelling of pre-conjugated RGD peptides took place in presence of [^76^Br]NH_4_Br in water, followed by the addition of 32% peracetic acid in MeCN. The mixture was stirred and heated at 80 °C for 10 min or at RT to test the temperature effect. To obtain the pre-conjugated RGD peptides, the coupling between the lysine of c(RGDfK) and *N*-succinimidyl 2, 6-dimethoxybenzoate was conducted in presence of DIPEA in DMF and the mixture was stirred at RT for 1 h. After quenching with acetic acid, the reaction mixture was diluted with water and purified by preparative RP HPLC. The coupling yields of [^76^Br]SBDMB with RGD peptides were over 70% and the specific activity, at the end of synthesis, for [^76^Br]BrDMB-E[c(RGDyK)]_2_ used for imaging studies, was 262 mCi μmol^−1^. The yield for ^76^Br labelling of pre-conjugated RGD peptides was over 60% at 80 °C using diluted peracetic acid. The yield increased when more concentrated peracetic acid was used at RT. The direct labelling with ^76^Br of no pre-conjugated RGD peptides with chloramine-T or peracetic acid showed lower yields and purities. In any case, high labelling yields were achieved under mild reaction conditions without the need for a trialkylstannyl leaving group.

## Design and strategies for ^86^Y marked peptides

**Fig. 12:**
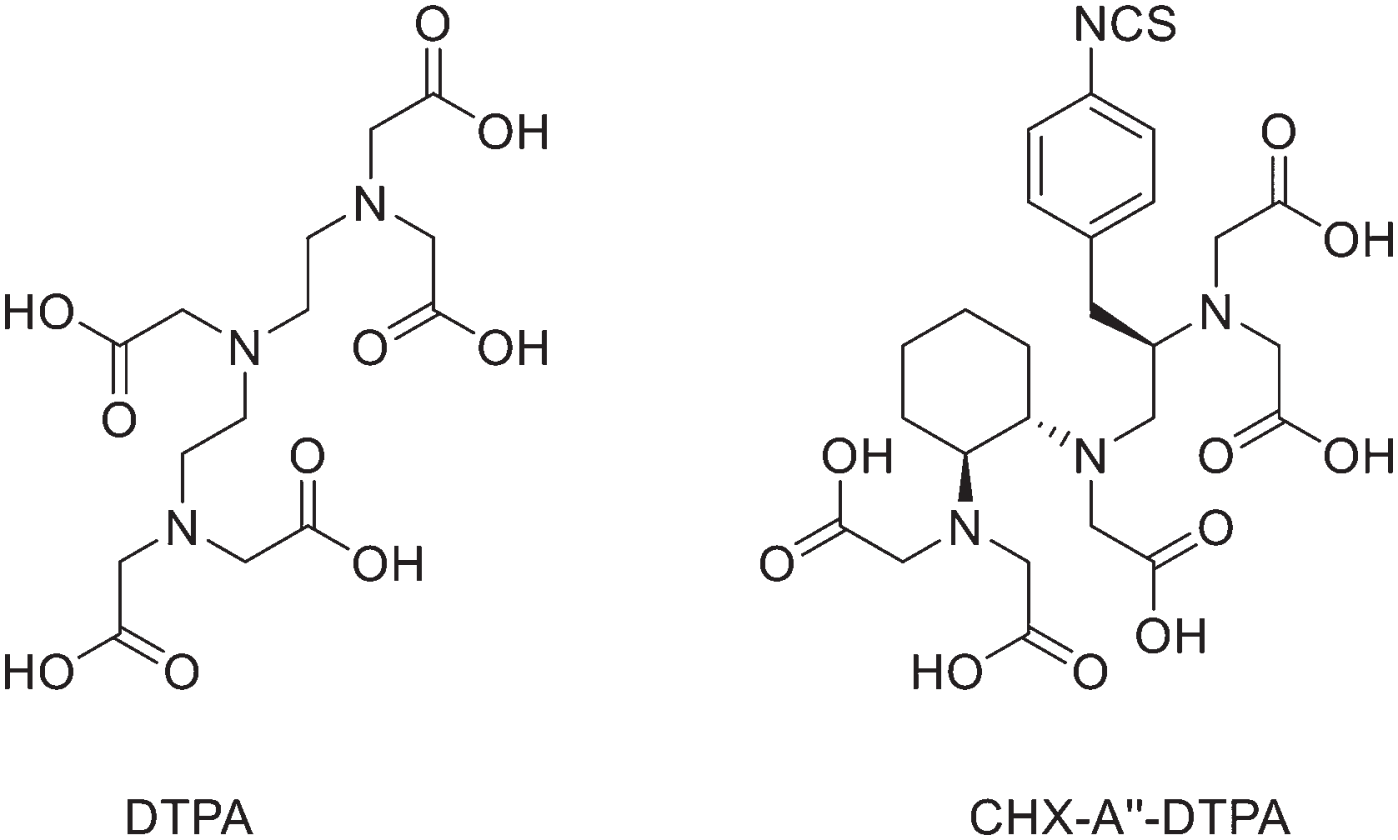
Structures of the parent compound DTPA and the isothiocyanate derivative of CHX-A′′ (CHX-A′′-DTPA).

^86^Y is a non-standard positron emitter with a half-life of 14.7 h, which has recently become popular since it is chemically equivalent to ^90^Y and the use of ^86^Y-labelled pharmaceuticals for quantitative PET imaging allows for patient-specific dosimetry before ^90^Y therapy. This transition metal ion exists as Y^3+^ and it is considered a hard acid that generates stable complexes with ligands containing hard donor atoms. For example, EDTA, DTPA and DOTA are the most common used BFCAs for the labelling of peptides with yttrium isotopes. Macrocyclic complexes based on the DOTA ligand are thermodynamically more stable than those based on the acyclic DTPA.^138^ A way to increase their stability is to increase the backbone rigidity of these complexes. For example, CHX-A′′-DTPA is a typical derivative of DTPA that bears a rigid cyclohexane group in the DTPA backbone (Fig. 12).

Clifford *et al.* developed an *N*-hydroxysuccinimidyl penta-*tert*-butyl ester derivative of CHX-A′′-DTPA with a glutaric acid spacer moiety linked to the N-terminal of the eight-amino acid somatostatin analogue octreotide and then radiolabelled with ^86^Y.^139^ For the conjugation, the N-terminus of the peptide was linked to the chelate by mixing with 1.5 equivalents of chelate in DMF. The reaction lasted a total of 6–8 h, and precipitation from diethyl ether afforded the crude product. The Boc and *tert*-butyl ester groups were removed using TFA/phenol/thioanisole/EDT/H_2_O and then purification by RP HPLC gave the final compound. Radiolabelling was achieved *via* the incubation of CHX-A′′-octreotide with ^86^YCl_3_ in 0.5 M NH_4_OAc at 25 °C. Radiochemical yields were > than 97% (0.1 mCi μg^−1^; 6.49106 MBq per mmole). This new chelating agent could be used both in a peptide synthesiser or in solution (after cleavage of the peptide from acid very sensitive resins in order to keep protection intact apart from the N-terminus) to modify a peptide at the N-terminus. Biddlecombe *et al.* evaluated the ^86^Y-radiolabelled peptide MP2346 (DOTA-(Pro^1^,Tyr^4^)-bombesin(1–14)) as a candidate for PET imaging of GRPR-positive tumours.^140^ MP2346 was previously synthesised and radiolabelled with ^111^In.^141^ The ^86^Y analogue showed good PET image quality in models of prostate cancer for the detection of GRPR-rich tumours.

More recently Bandara *et al.*^143^ developed the [RGD-Glu-6AhxRM2] peptide to target αvβ3 receptors (where RGD: Arg–Gly–Asp; Glu: glutamic acid; 6-Ahx: 6-amino hexanoic acid; RM2: (d-Phe–Gln–Trp–Ala–Val–Gly–His–Sta–Leu–NH_2_)) that was linked to DOTA to obtain [RGD-Glu-[DO3A]-6-Ahx-RM2] (previously described by Reynolds and co-workers^142^) and radiolabelled it with ^86^Y and ^90^Y to obtain a new “matched-pair” tracer conjugate. In general, ^86^Y does not represent the perfect isotope for PET imaging since more than 60% of its β^+^ decay processes go along with the emission of gamma rays, which could give background signals. New approaches are being developed to implement background corrections to ^86^Y PET images to reduce these interference problems.^144^

## Perspective and conclusion

Peptide-based PET probes are relatively small tracers with high affinity and specificity to specific cellular targets, appropriately engineered for acting as PET probes for diagnosis and/or therapy (*i.e.* theranostic agents). It is not surprising that, due to the pivotal role of peptides/cellular receptors interactions, the development of radiolabelled PET probes based on small peptides has been of interest for decades. Peptide-based probes show many advantages respect to common probes based on antibodies: peptides are cheaper and more suitable for site-specifically chemically modification to allow radiolabelling with potentially any radionuclide. The radioisotope can be covalently linked to the peptide-probe or the probe could be functionalised with another complexing agent for the coordination of the radioisotope; all these features make them very attractive for clinical applications. Moreover, the range of labelling options and ways to modify and optimize the pharmacokinetic properties of such compounds makes this class of tracers very attractive for the development of radiopharmaceuticals for the diagnosis/therapy of oncological diseases. Most recent advantages in this field have both expanded the scope of well-established techniques and expanded PET imaging into unstudied areas. The goal of this review was to report the most used and innovative synthetic approaches for the radiolabelling of peptide-based PET probes. As reported in this review, to emulate and facilitate real-life conditions of radiolabelling, *i.e.*, radiopharmacies in hospitals, the research into this field has mainly focused on replacing harsh reaction conditions with simple and mild reaction conditions. This effort has been successful in several ways, such as utilizing click chemistry to quickly attach prosthetic groups (*e.g.*, for ^18^F) and synthesising new chelating moieties that react at room temperature and neutral pH in as little as 10 min, such as the THP for ^68^Ga. Over the past years, a large interest has begun around this radiolabelling strategy, resulting in metallic radionuclides and the development of new chelators – THP, TRAP and FSC for ^68^Ga, DFO for ^89^Zr, or sarcophagines for ^64^Cu, and linkers for an effective conjugation between metals and biomolecules, at the forefront research in the field. The interest in radiolabelled peptides is currently due to high compatibility with many protein structures overexpressed in several diseases, including cancer. The integrin αvβ3, PSMA, as well as cMet receptors targeting probes are very attractive molecular targets due to their overexpression within a range of cancers regulating growth, proliferation, and metastasis. In nuclear medicine research, gallium-68, DOTA-based chelators, and amino acid linkers are currently dominating the research of new potential diagnostic and imaging agents.^145^ In centres, where ^68^Ga-compounds cannot be used due to gallium unavailability, yttrium-86 or zirconium-89 are normally employed. All the other examples of radionuclides reported in this review are either in the preclinical or clinical research phase, and with this the review it is hoped to assist scientists in the field of peptide PET probe labelling by reporting all the cutting-edge technology that is currently available for the chemical modification of peptides in order to make them suitable for PET imaging.

## Abbreviation

Acm: Acetamidomethyl
Ahx: 6-Aminohexanoic acid
Aoc: 8-Aminooctanoic acid
DCM: Dichloromethane
Dde: *N*-(1-(4,4-Dimethyl-2,6-dioxocyclohexylidene)ethyl)
DIC: *N*,*N*′-Diisopropylcarbodiimide
DIEA: *N*,*N*-Diisopropylethylamine
DIPEA: *N*,*N*-Diisopropylethylamine
DMA: *N*,*N*-Dimethylacetamide
DMF: Dimhetylformamide
DTPA: Diethylenetriaminepentaacetic acid
EDC: 1-Ethyl-3-(3-dimethylaminopropyl)carbodiimide
EtOH: Ethanol
HATU: 1-[Bis(dimethylamino)methylene]-1*H*-1,2,3-triazolo[4,5-*b*]pyridinium 3-oxid hexafluorophosphate
HBTU: *N*,*N*,*N*′,*N*′-Tetramethyl-*O*-(1*H*-benzotriazol-1-yl)uronium hexafluorophosphate, *O*-(benzotriazol-1-yl)-*N*,*N*,*N*′,*N*′-tetramethyluronium hexafluorophosphate
HEPES: 2-[4-(2-Hydroxyethyl)piperazin-1-yl]ethanesulfonic acid
HFIP: 1,1,1,3,3,3-Hexafluoroisopropanole
HOAt: 1-Hydroxy-7-azabenzotriazole
HOBt: Hydroxybenzotriazole
MeCN: Acetonitrile
MeOH: Methanol
Na_2_EDTA: Disodium ethylenediaminetetraacetic acid
NMP: *N*-Methyl-2-pyrrolidone
Pbf: 2,2,4,6,7-Pentamethyl-2,3-dihydrobenzofuran-5-sulfonyl
PyBOP: Benzotriazol-1-yloxytripyrrolidinophosphonium hexafluorophosphate
SNHS: *N*-Hydroxysulfosuccinimide
TBAF: Tetrabutylammonium fluoride
*t*-Boc: *Tert*-butyloxycarbonyl
*t*-Bu: *Tert*-butyl
TEA: Triethylamine
TFA: Trifluoroacetic acid
THF: Tetrahydrofuran
THPTA: Tris(3-hydroxypropyltriazolylmethyl)amine
TIBS: Tris(2-methylpropyl)silane
TSTU: *O*-(*N*-Succinimidyl)-*NN*, *N*′,*N*′-tetramethyluronium tetrafluoroborate

## Conflicts of interest

There are no conflicts to declare.

## Supplementary Material
